# Percolation thresholds for photonic quantum computing

**DOI:** 10.1038/s41467-019-08948-x

**Published:** 2019-03-06

**Authors:** Mihir Pant, Don Towsley, Dirk Englund, Saikat Guha

**Affiliations:** 1 Department of Electrical Engineering and Computer Science, MIT, Cambridge, MA 02139 USA; 2 Quantum Information Processing group, Raytheon BBN Technologies, 10 Moulton Street, Cambridge, MA 02138 USA; 3 College of Information and Computer Sciences, University of Massachusetts, Amherst, MA 01003 USA; 4 College of Optical Sciences, University of Arizona, 1630 E University Blvd, Tucson, AZ 85719 USA

## Abstract

Despite linear-optical fusion (Bell measurement) being probabilistic, photonic cluster states for universal quantum computation can be prepared without feed-forward by fusing small *n*-photon entangled clusters, if the success probability of each fusion attempt is above a threshold, ${\mathrm{\lambda }}_{\mathrm{c}}^{(n)}$. We prove a general bound ${\mathrm{\lambda }}_{\mathrm{c}}^{(n)} \ge 1/(n - 1)$, and develop a conceptual method to construct long-range-connected clusters where ${\mathrm{\lambda }}_{\mathrm{c}}^{(n)}$ becomes the bond percolation threshold of a logical graph. This mapping lets us find constructions that require lower fusion success probabilities than currently known, and settle a heretofore open question by showing that a universal cluster state can be created by fusing 3-photon clusters over a 2D lattice with a fusion success probability that is achievable with linear optics and single photons, making this attractive for integrated-photonic realizations.

## Abstract (web summary)

Universal cluster states for quantum computing can be assembled without feed-forward by fusing n-photon clusters with linear optics if the fusion success probability is above a threshold p. The authors bound p in terms of n and provide protocols for n = 3 clusters requiring lower fusion probability than before.

## Introduction

Optical qubits are a promising candidate for scalable universal quantum computing because of the relative ease of generating photons, fabricating photonic circuits^1–3^, and low decoherence rates. Assembling a universal photonic cluster state, e.g., by fusing small entangled clusters using linear optical interferometers into progressively larger ones, is limited by probabilistic operations because of the limitations of linear optics. For example, a linear–optical circuit for a two-qubit Bell-state measurement (BSM)—the smallest primitive to “fuse” two cluster states—can succeed with at most probability 1/2 = 0.5^4,5^. However, since photons are the base resource to encode qubits anyway, it is reasonable to allow photons to be used as an ancilla resource to boost the success probability of linear–optic BSMs^6–8^. The maximum known BSM success probability attainable using linear optics, boosted by injecting eight ancilla single photons, is 25/32 = 0.78125^8^. It is not known if a higher BSM success probability is possible with linear optics boosted with more single photons. Very little is understood about similar success-probability limits associated with linear–optical realizations of larger (three or more qubits) projective measurements, and those for creating small photonic entangled clusters^9^. The lack of our understanding of the maximum efficiency in creating universal photonic cluster states goes back to the mathematical structure of manipulations of multiphoton entangled states using linear optics and photon detection being complex, as evidenced by the hardness of boson sampling^10^.

Linear optical quantum computing (LOQC) in its widely known circuit-model form—coined by the seminal KLM paper^11^—employs single-photon dual-rail qubits (|**0**〉 ≡ |10〉, |**1**〉 ≡ |01〉), probabilistic linear–optical gates, single-photon detection, and measurement-induced feedforward. Even though KLM’s scheme in principle can do universal quantum computing, the overheads are astronomically high. Many variants of KLM were proposed^12–14^ that use entangled ancillas to reduce the overheads, but the hardness was pushed into creating those ancillas. Schemes for efficient generation of these ancillas from simpler resources such as single photons remain unknown. Furthermore, the need for detection-induced feedback creates additional experimental issues. This resulted in LOQC being largely disfavored in lieu of other qubit technologies, such as superconducting and trapped-ion qubits.

**Fig. 1:**
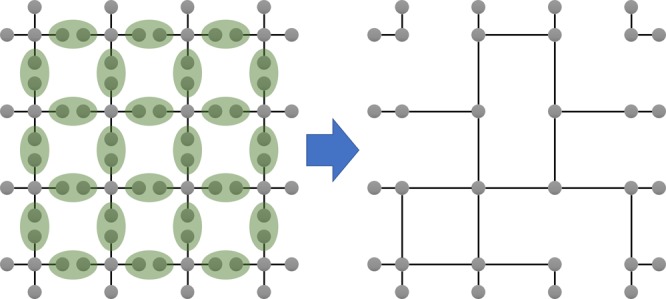
Creating a percolated square lattice with fusion. Fusing five-photon star clusters into a percolated 2D square lattice is possible with two-qubit fusion operations, if each fusion attempt succeeds with the probability more than 0.5, the bond-percolation threshold of the lattice^15^

**Fig. 2:**
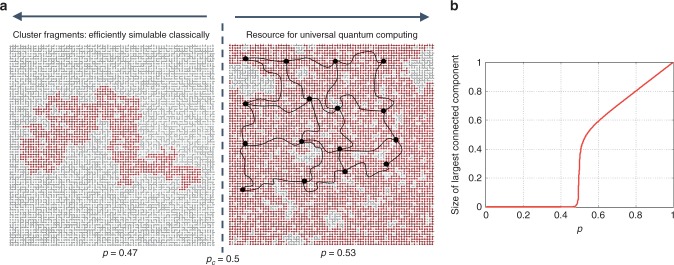
Bond-percolation threshold of a square lattice. **a** Each bond in a regular lattice of *m* bonds is activated with probability *p*. If *p* < *p*_c_ (the subcritical regime), where *p*_c_ is the bond-percolation threshold, the size of the largest connected component is *O*(log *m*). At *p* = *p*_c_, a sharp transition occurs, and for 1 ≥ *p* > *p*_c_, the size of the largest connected component is *O*(*m*). In this (supercritical) regime, *O*(*m*) criss-crossing edge-disjoint paths (percolation highways) exist from left to right and from top to bottom^17^, using which one can identify a logical lattice whose nodes are the intersection points of the highways, and edges are paths connecting those nodes. **b** Size of the largest connected component, expressed as a fraction of *m*, as a function of *p* ∈ [0, 1]

Kieling, Rudolph, and Eisert proposed a cluster-model version of LOQC^15^, which mitigates these issues. Their scheme places *d* + 1 photon “star” clusters at nodes of a regular lattice G with degree-*d* nodes and stitches them together using two-photon fusions (BSMs) along the lattice edges (see Fig. 1). If the success probability of each fusion attempt *λ* is above the bond percolation threshold *p*_c_(G) of the underlying lattice G, the resulting cluster state will be a long-range-connected (random) subgraph of G, which can then be renormalized into a universal cluster state by identifying a subarray of nodes in the percolated lattice—the intersection points of vertical and horizontal “percolation highways”^16,17^—to serve as the nodes of a logical universal lattice and identifying paths connecting them through other nodes of the percolated subgraph as edges of this logical lattice (see Fig. 2a). The inspiration behind this model is that if small *d* + 1 photon clusters can be created, and a lattice can be identified whose bond- percolation threshold is below a practically attainable fusion success probability, then the clusters can be linear–optically fused into a large universal cluster state in a one-shot (or “ballistic”) fashion, with no detection-induced feedforward. In order to produce conservative estimates for the fusion success probability required to produce a renormalizable cluster state, this work disregarded the “failure modes” of the fusion attempts, i.e., the leftover cluster states when fusion attempts fail.

Significant progress in this model of LOQC came recently from Rudolph and collaborators, who by paying closer attention to the failure modes, and a clever design of 3D lattices with a low-percolation threshold, e.g., the diamond^18^ and the pyrochlore lattice^19^, were able to show that ballistic creation of a universal cluster state is possible starting with a large supply of three-photon-entangled (GHZ state) clusters, with two-photon fusions that succeed with a probability ≈0.625. Since 0.625 < 25/32 = 0.78125, the best-known success probability attainable with linear optics boosted with unentangled single-photon ancillas^8^, this gave the first realizable ballistic construction of a universal cluster if three-photon GHZ clusters were available as a starting point.

The results of Gimeno-Segovia et al. and Zaidi et al.^18,19^ on ballistic (feedback-free) creation of universal clusters, in conjunction with a host of progress in experimental proposals to directly create small photonic entangled clusters using quantum emitters^20–22^ (rather than starting with single photons and fusing them probabilistically using heralded methods into larger clusters), have resurrected the optimism in scalable optical quantum computing. However, the fundamental limits of efficiency and resource overhead associated with this philosophy—that of direct generation of a universal cluster state using small photon clusters and linear optics—remain unanswered. Refs. ^18,19^ present two (clever, yet ad hoc) examples of constructions that use three-photon clusters as the starting point.

The goal of this paper is to explore this fundamental limit. We address the following general question:

Given an unlimited supply of *n*-photon-entangled dual-rail-basis clusters, and repeated use of a linear–optical circuit that realizes two-qubit fusion with probability of success λ, but with no restrictions on how the clusters to be fused are aligned in space and time, what is the minimum value ${\lambda }_{\mathrm{c}}^{(n)}$ of each fusion’s success probability that will allow for ballistic creation of a universal cluster state?

Before we proceed, it should be clear that the two examples we discussed above—one in Fig. 1^15^ and that in ref. ^18^—establish the following bounds:

1 $${\lambda }_{\mathrm{c}}^{(5)} \le 0.5,$$

and

2 $${\lambda }_{\mathrm{c}}^{(3)} \,\, \lesssim \,\, 0.625,$$

where we use ≲ sign instead of ≤ in Eq. (2), since the 0.625 threshold was an approximate numerical estimation of the threshold using a small-sized diamond lattice^18^.

We focus on Bell-measurement-based fusion operations. Although Browne and Rudolph’s original paper that coined the term “fusion”^13^ also proposed an alternative linear optical fusion circuit (called fusion-I), subsequent work has focussed on Bell-measurement-based fusion (fusion-II of ref. ^13^) because of its natural loss tolerance and recent progress in boosting its success probability by injecting unentangled single-photon ancillas^8^.

In this paper, we prove that starting with *n*-photon clusters, a fusion success probability of at least 1/(*n*−1) is required to create a resource state for universal quantum computation without feedforward. We map a planned set of fusion operations on photon pairs among a regular spatial array of *n*-photon clusters—the cluster-fusion lattice, G_1_—to an instance of bond percolation on a logical lattice G_2_. We use this map to find new constructions to produce universal photonic cluster states starting with three-photon cluster states as the initial resource, without feedforward, that require a lower fusion success probability than was previously known. Finally, we settle a heretofore open question by showing that a universal cluster state can be created by fusing three-photon clusters over a 2D lattice with a fusion success probability that is achievable with linear optics and single photons, making this attractive for integrated-photonic realizations.

## Results

### Summary of the main results

The main results in this paper are as follows:

1. We prove a general lower bound ${\lambda }_{\mathrm{c}}^{(n)} > 1/(n - 1)$, and provide evidence in favor of our conjecture that it is tight (achievable as closely as we wish).
2. We develop a systematic graphical construction of a universal cluster, wherein ${\lambda }_{\mathrm{c}}^{(n)}$ becomes the usual bond-percolation threshold of a logical graph that we specify, while accounting for the fusion failure modes. This is a key realization that was missing in both Kieling et al.’s construction^15^ as well as in refs. ^18,19^. This not only enabled us to readily generate achievable thresholds (upper bounds on ${\lambda }_{\mathrm{c}}^{(n)}$) using existing literature on bond percolation, but also helped us to prove the aforesaid lower bound. We believe that this logical construction also naturally generalizes to future extensions that may employ multi-qubit fusions (projective measurements). We describe this construction systematically in the section “Reconciling prior results in a new framework”, and present several improved thresholds using this new formalism. However, we develop a fully axiomatic construction of this logical graph for the most general case if left open for future work.
3. Using our logical construction, we prove ${\lambda }_{\mathrm{c}}^{(3)} \le 0.5898$ as an achievable threshold, which improves upon the best existing result in ref. ^18^. As a by-product of our logical construction allowing a standard bond-percolation interpretation, we were able to reinterpret the construction of ref. ^18^ in our formalism and used Newman and Ziff’s efficient Monte Carlo algorithm to refine their threshold (2) to ${\lambda }_{\mathrm{c}}^{(3)} \le 0.627$.
4. We settle a heretofore open question posed by Rudolph and collaborators^18,19,23^ by showing that a universal cluster can be created by linear–optically fusing three-photon GHZ clusters over a 2D (brickwork) lattice with fusion success probability 0.746, which is below 25/32, the maximum known fusion success probability achievable with a single-photon-boosted linear–optic scheme^8^, making this attractive for integrated-photonic realizations.

In the next section, we will begin with a discussion of the high-level problem setup and the constraints therein, following which we will present a logical technical progression leading to our main results and the key intuitions. Detailed derivations and technical graphical constructions not central to following the main arguments in the paper are presented in the Supplementary Note 2.

### Generality and constraints of our problem setup

**Fig. 3:**
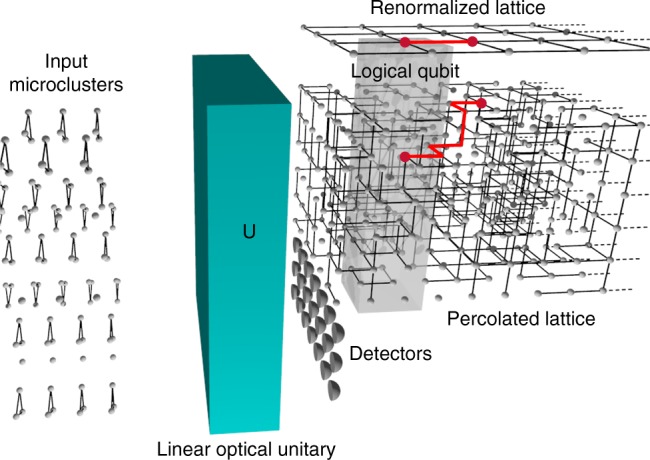
Ballistic photonic cluster state generation for quantum computing. A steady stream of entangled clusters of *n*-photons or less (*n* = 3 shown) is incident on a linear–optical interferometer (i.e., a multimode unitary transformation **U**), which produces an entangled cluster of photons at its output. If the percolation condition is met, the output can be renormalized into a fully connected logical cluster for universal cluster-model quantum computing. The detector outputs are not used to herald whether or not the undetected outputs are in a universal cluster state. They always are. The detector outputs carry complete information on the location of missing edges in the random graph, and thus how to renormalize it

Figure 3 and its caption explains our high-level problem setup. It follows from our discussion above that we are limiting ourselves to only those multimode linear–optical unitaries **U** that can be pieced together using a sequence of two-qubit linear–optic BSMs. Our problem formulation, however, subsumes the special cases of the constructions in refs. ^15,18,19^. Furthermore, our formulation allows for yet-undiscovered fusion circuits that may be boosted with up to *n*-photon entangled clusters (which may attain a success probability greater than the currently best known, 25/32). It also admits generalizations to multi-qubit fusion, i.e., *m*-qubit GHZ-state projections, the theory behind optimal linear–optical realizations of which it remains not well understood.

The detectors shown in Fig. 3 are those corresponding to the linear–optical fusion gates pushed (deferred) all the way to the right with no loss of generality. Similarly, any photons or photon clusters used to boost any fusion gate are pushed to the left of **U**. This way, one can envision the cluster preparation as a multimode linear optical circuit presented with individual photons and photon clusters as input, and producing (deterministically) a renormalizable universal cluster state as its output. The individual detector outputs are random, but if we design **U** in a way that the percolation condition is met, the undetected outputs of **U** would, with high probability, carry a random instance of a universal long-range-connected (percolated) cluster and the vector of detector outputs would simply tell us what cluster state got created on the undetected outputs, and thus give us the necessary data to compute criss-crossing edge-disjoint paths through the percolated lattice to renormalize it into a universal logical cluster state^24^.

### General lower bounds

In this section, we present an intuitive explanation of our lower bound on ${\lambda }_{\mathrm{c}}^{(n)}$. A detailed formal proof is provided in Supplementary Note 1.

We have the following general lower bound:

3 $${\lambda }_{\mathrm{c}}^{(n)} \ge \frac{1}{{n - 1}},\forall n \ge 2.$$

Proof sketch—starting with *n*-photon clusters, and any sequence of fusion attempts, the resulting instance of the logical graph is a bond-percolation instance, with bond success probability *λ*, on some graph of maximum degree *n*. Of all infinite graphs of maximum degree *n*, the minimum bond-percolation threshold is that of the degree-*n* Bethe lattice, and equals 1/(*n*−1). This implies that if *λ* < 1/(*n*−1), the percolation condition cannot be met no matter what. Hence, ${\lambda }_{\mathrm{c}}^{(n)} \ge 1/(n - 1)$.

Since the Bethe lattice is a tree and since trees are not a universal resource, this does not prove 1/(*n*−1) to be an achievable threshold for universal cluster creation (see Supplementary Note 3 for more details). However, we conjecture (and provide evidence in its favor) that *λ* = [1/(*n*−1)] + *ε* with any *ε* > 0 that may suffice to create a universal cluster state. This conjecture is supported by the fact that starting with *N* copies of photon clusters of size *n* and probabilistic operations that succeed with any probability greater than 1/(*n*−1), it is possible to obtain an *O*(*N*)-sized tree graph (detailed in Supplementary Note 3). We also present a random graph construction, which provides intuition for why probabilistic operations on photon clusters of size *n* may provide enough connectivity to obtain a large connected cluster when *λ* > 1/(*n*−1) (Supplementary Note 3).

We also show that if *m*-qubit fusions are used to fuse *n*-qubit clusters for *m* ≥ 2, the optimum threshold on the fusion success probability required to generate a universal cluster ballistically must satisfy

4 $${\lambda }_{\mathrm{c}}^{(n,m)} \ge \frac{1}{{(n - 1)(m - 1)}},\forall n \ge 2,m \ge 2.$$

Very little is known about linear–optical circuits for *m* > 2 qubit fusion^9^ (e.g., projecting three qubits to one of the eight orthogonal three-qubit GHZ states), their associated success probabilities, and various failure outcomes. Thus, it remains unclear if the above bound on ${\lambda }_{\mathrm{c}}^{(n,m)}$ is tight.

### Reconciling prior results in a new framework

Our next contribution is a reinterpretation of the constructions in refs. ^15,18,19^ as a standard bond-percolation problem on a logical lattice, which lets us identify (and sharpen) the 0.627 threshold found in ref. ^18^ as the standard bond-percolation threshold of a 3D-modified (10,3)-b lattice^25^.

**Fig. 4:**
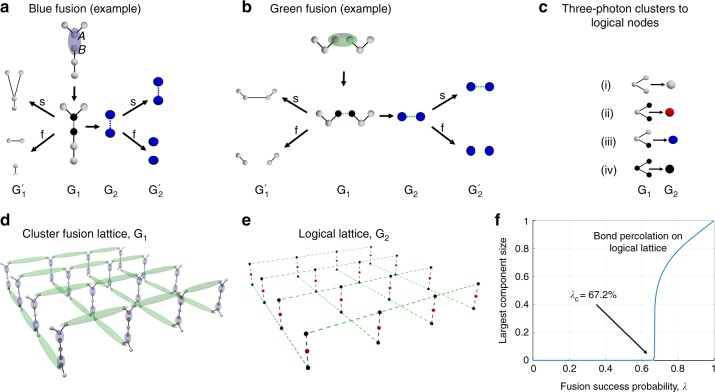
Mapping two-photon fusions with many three-photon clusters to bond percolation on a logical graph. **a**, **b** Examples of two types of two-photon fusion operations, and their post-measurement states for success and failure outcomes. The linear–optical circuits for these (blue and green) fusions are shown in Figs. 2 and 3 of ref. ^18^, respectively. From a graph connectivity point of view, two-photon fusion can be interpreted as coloring the measured photons black, and if the fusion succeeds, drawing a new bond between the black photons. **c** We map three-photon clusters to nodes of a logical graph of color based on how many photons in the cluster are left unmeasured. **d** An example of a cluster-fusion lattice G_1_ with both kinds of fusions, and (**e**) the corresponding logical graph G_2_. **f** The bond-percolation curve and threshold (*λ*_c_ ≈ 0.672) of G_2_. If each fusion attempt in (**d**) succeeds with probability *λ* > *λ*_c_, it results in a random graph ${\mathrm{G}}_2^\prime$ which is a giant-connected component (GCC) consisting of red nodes of G_2_. This implies the creation of a random photonic cluster state ${\mathrm{G}}_1^\prime$, which is a GCC of G_1_ comprising the unmeasured photons—a resource renormalizable for universal cluster-model photonic quantum computing

We first describe the mapping of a spatially regular arrangement of fusion attempts among photons in *n*-photon clusters—the cluster-fusion lattice G_1_—to a logical lattice G_2_, each of whose nodes correspond to an *n*-photon cluster. We then show that the standard bond-percolation threshold of G_2_ equals the threshold on the success probability of fusion attempts in G_1_ exceeding, which generates, with high probability, a long-range-connected (random) photonic lattice ${\mathrm{G}}_1^\prime$ among the unmeasured photons in G_1_, which is renormalizable for universal quantum computing. The mapping is illustrated via an example shown in Fig. 4. We will restrict, for simplicity, to *n* = 3 photon-line clusters as the initial resource to explain the mapping. But the G_1_-to-G_2_ mapping we describe below, holds for clusters of any size *n* and shape.

G_1_ represents the pattern of pairwise fusion attempts we intend to perform on a subset of photons across many three-photon clusters. To construct G_1_, we begin with (1) a (two- or higher-dimensional) spatial arrangement of three-photon line clusters, (2) identifying pairs of photons (belonging to distinct clusters), upon which fusions will be attempted, and (3) one of two types for each of those fusions: blue or green, linear optical circuits to realize, which are shown in Figs. 2 and 3 of ref. ^18^, respectively. Success and failure outcomes of an example green and blue fusion are shown in Fig. 4a, b.

We thus form the cluster-fusion lattice G_1_:

- The nodes of G_1_ are photons, colored white or black: referring to whether the photons will be left unmeasured vs. will be measured (and hence destroyed) in fusion attempts.
- The edges of G_1_ are of three kinds: black corresponding to the pre-existing entanglement between photons within a three-photon cluster, and, blue and green (dashed) edges connecting black nodes corresponding to two types of (planned) fusion attempts.

Now, we construct the logical graph G_2_, each of whose nodes correspond to one three-photon cluster of G_1_. The color of a node of G_2_—white, red, blue, and black, is determined based on the number of black (measured) photons in the corresponding photonic cluster in G_1_: (i) zero, (ii) one, (iii) two, or (iv) three, respectively (see Fig. 4c). There are two kinds of edges in G_2_: blue or green, corresponding to the two types of fusion attempts.

We impose four conditions on the graphs we consider, which are not required for the above G_1_ → G_2_ mapping, but needed for our percolation-threshold equivalence to hold. Each condition can be stated either for G_1_ or G_2_.

- Condition 1: Any loop in G_2_ must contain at least three nonblack nodes.
- Condition 2: One endpoint of a blue-dashed edge in G_1_ must be the degree-2 (middle) node of a three-photon cluster. We will call this node A. The other end node is a degree-1 (leaf) node of a three-photon cluster. We call this node B.
- Condition 3: Both endpoints of a green-dashed edge in G_1_ must be degree-1 (leaf) nodes of two three-photon clusters.
- Condition 4: For each cluster in G_1_, all whose photons are measured in fusion attempts, there is at least one cluster with one unmeasured photon at a constant distance (number of hops) away. Translated to G_2_, this implies that every black node has a nonblack node at a constant distance from it (e.g., one bond away for the example shown in Fig. 4e).

We now describe the action of the blue and green fusion operations on photonic cluster states. In the following discussion, we will use “cluster edge” and “cluster node” to refer to edges and nodes in a photonic cluster state, to avoid any confusion with the logical graph. A “cluster edge” refers to entanglement between neighboring photons (e.g., black edges in G_1_) and not fusion attempts (e.g., blue and green-dashed edges of G_1_). A “cluster node” is a photon.

After the fusion attempts in G_1_ have been made, all the black cluster nodes in G_1_ are destroyed. If a fusion attempt is successful, then additional cluster edges (depicting a newly created entanglement) appear among cluster nodes that were former neighbors of the measured (black) cluster nodes. Each fusion attempt succeeds or fails with probability *λ* or 1−*λ*, respectively. Depending upon the success–failure outcomes of all the fusion attempts, a new photonic lattice is created—a random graph state ${\mathrm{G}}_1^\prime$—whose cluster nodes are the white cluster nodes (unmeasured photons) of G_1_.

In order to describe the ${\mathrm{G}}_1 \to {\mathrm{G}}_1^\prime$ mapping, we need to describe the success and failure outcomes of both kinds of fusion. To do so, we need a few additional definitions:

- Cluster edge parity (of a pair of cluster nodes): The parity of a cluster edge is 1 if the cluster edge exists, and 0 otherwise.
- Neighborhood inversion (on a cluster node): A unitary action on a cluster node in a photonic cluster state, which flips the cluster edge parity between every pair of neighbors of the cluster node. So, some cluster edges can be deleted, and new cluster edges can be created.

Let us now describe the action of the two fusion operations. Either type of fusion attempt destroys the two photons fused, regardless of whether the fusion succeeds.

- Blue fusion: Fig. 4a shows an example of a blue fusion on a pair of photons in two three-photon clusters. The blue fusion is asymmetric across the two cluster nodes it acts upon. Let us label the measured cluster nodes A and B. If the fusion succeeds, it flips the cluster edge parity between every neighboring pair of A and B. In other words, if one neighboring cluster node of A and one neighboring cluster node of B had no direct cluster edge between them prior to the fusion attempt, after successful fusion, they get a direct cluster edge between them. Also, if a neighbor of A and a neighbor of B had a direct cluster edge between them before fusion, this cluster edge is removed after successful fusion^18^. Condition 1 above prevents the latter to ever happen, i.e., we never attempt to fuse two cluster nodes whose neighbors had a direct cluster edge prior to the fusion attempt. If a blue fusion fails, we first perform a neighborhood inversion on cluster node A, i.e., we flip the cluster edge parity between every pair of neighbors of cluster node A and then remove both cluster nodes A and B from the graph.
  If the blue fusion shown in Fig. 4a succeeds, we obtain a four-photon cluster state. If it fails, we perform a neighborhood inversion on cluster node A and remove both measured cluster nodes, leaving the two cluster fragments with two photons in each. Condition 2 above ensures that one of the two cluster nodes for blue fusions is a leaf cluster node of a cluster. This is the B cluster node, which does not undergo neighborhood inversion after a fusion attempt failure.
- Green fusion: The green fusion, shown in an example in Fig. 4b, has the same behavior as the blue fusion when it succeeds. As in the case of the blue fusion, we never fuse two cluster nodes whose neighbors already have a direct cluster edge, due to Condition 1 we imposed above. In the case of failure, we simply remove the two measured cluster nodes and all their cluster edges. The green fusions are symmetric, and as stated above in Condition 3, always used between a pair of leaf cluster nodes of two clusters.

We introduced above the (deterministic) lattice G_1_ denoting cluster-fusion attempts, and mapped it to a (deterministic) logical lattice G_2_, whose edges denote fusion attempts. The probabilistic success–failure fusion outcomes map G_1_ to a (random) photonic lattice ${\mathrm{G}}_1^\prime$ among the unmeasured photons, and map G_2_ to a (random) lattice ${\mathrm{G}}_2^\prime$, which is a standard bond-percolation subgraph of G_2_ where each edge is missing (independent of the other edges) with probability 1−*λ*.

Under a given success–failure fusion outcome pattern, we will now argue that if ${\mathrm{G}}_2^\prime$ has a giant-connected component (GCC), which will happen if *λ* > *p*_c_(G_2_), the bond-percolation threshold of G_2_, then ${\mathrm{G}}_1^\prime$—the photonic lattice involving the unmeasured nodes—will also have a GCC, which is a lattice renormalizable for universal cluster-model quantum computing.

The black nodes in G_2_ disappear after fusion attempts (they have no photons left in them), but help provide connections between (the unmeasured photons within) the nonblack nodes.

If *N* is the number of nodes in G_2_, bond percolation on G_2_, i.e., *λ* > *p*_c_(G_2_) ensures that ${\mathrm{G}}_2^\prime$—the random bond-percolation instance (subgraph) of G_2_—contains a unique GCC, i.e., a single subgraph of ${\mathrm{G}}_2^\prime$ has *O*(*N*) nodes (black and nonblack nodes combined). However, since every black node has a nonblack node at a constant distance from it (Condition 4 above), the probability of a nonblack node being connected to the nearest black node is a constant. Therefore, the presence of *O*(*N*) black nodes in the GCC implies the presence of *O*(*N*) nonblack nodes in the GCC, and hence, there must be *O*(*N*) nonblack nodes in the GCC of ${\mathrm{G}}_2^\prime$.

Given any success–failure pattern of all the fusion attempts, two unmeasured photons, i.e., nodes of ${\mathrm{G}}_1^\prime$, have a connected path in ${\mathrm{G}}_1^\prime$ if and only if the corresponding white nodes in G_1_ have a connected path, via black edges and successful fusion (green or blue-dashed) edges. This can be seen through the examples shown in Fig. 4a, b. This, and the fact that nonblack nodes of G_2_ map to unmeasured (white) nodes of G_1_, a GCC with *O*(*N*) nonblack nodes in ${\mathrm{G}}_2^\prime$ implies the presence of a GCC of *O*(*N*) (white) nodes in ${\mathrm{G}}_1^\prime$.

Hence, if *λ* > *p*_c_(G_2_), the leftover photonic lattice ${\mathrm{G}}_1^\prime$ will have a GCC, which is a lattice renormalizable for universal cluster-model quantum computing. For the G_2_ in Fig. 4e, we numerically get *p*_c_(G_2_) ≈ 0.672. See plot in Fig. 4f. This establishes that ${\lambda }_{\mathrm{c}}^{(3)} \le 0.672$.

It is instructive to note here that if the logical graph is composed only of black and red nodes, the post-fusion random photonic cluster state ${\mathrm{G}}_1^\prime$ is a random subgraph of G_3_: the effective lattice formed by the red nodes alone (each of which are three-photon clusters with one photon unmeasured). The nodes of G_3_ are the red nodes of G_2_. In the example shown in Fig. 4e, even though G_2_ is a nonplanar two-layer lattice, ${\mathrm{G}}_1^\prime$ is a random subgraph of G_3_, a planar (2D) square lattice. All the logical graph examples in this paper will have the aforesaid property, i.e., composed of black and red nodes only.

Further intuition regarding the development of the above construction, and an alternative way to view the generation of ${\mathrm{G}}_1^\prime$ as a modified site–bond percolation on G_1_ directly, are provided in Supplementary Note 2.

**Fig. 5:**
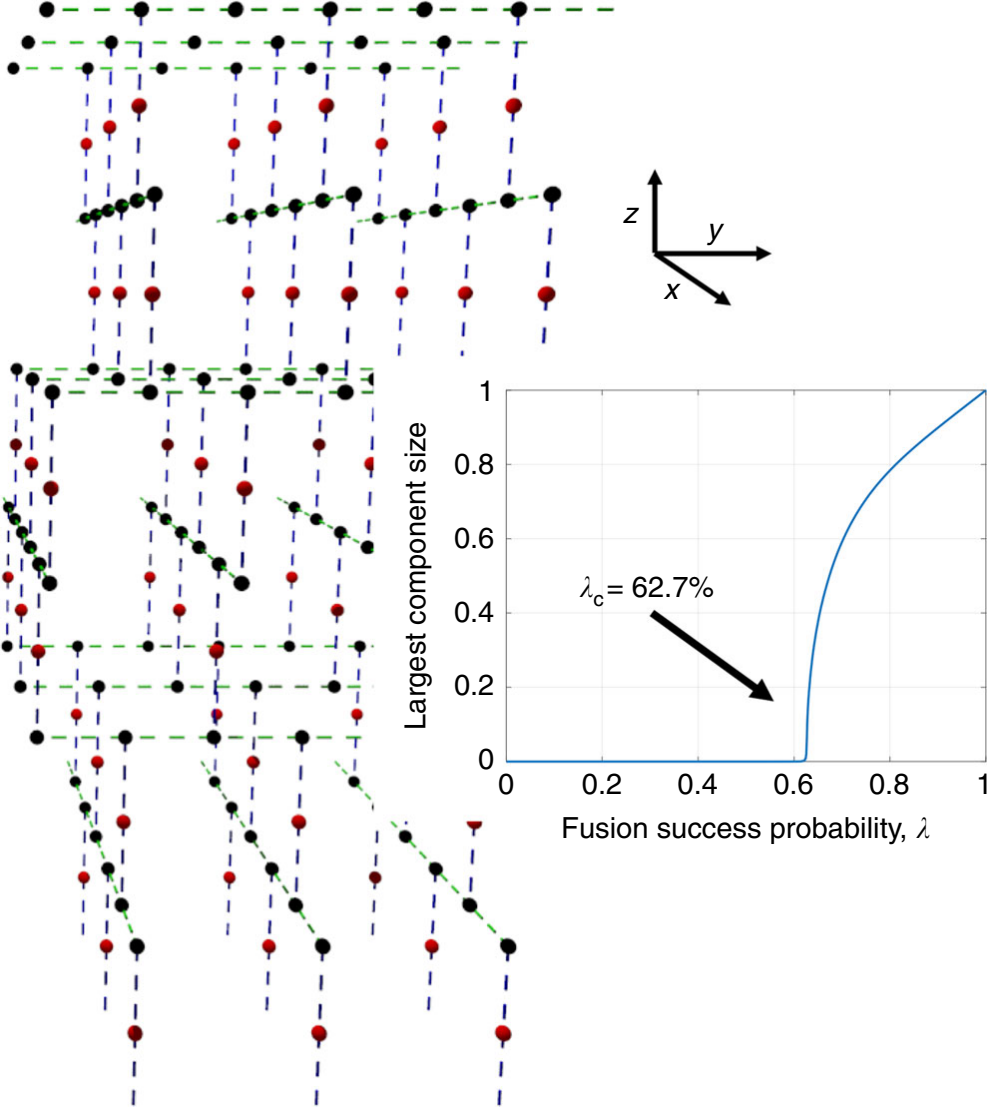
Bond percolation on the modified (10,3)-b lattice. The (*x*, *y*)-plane layers of parallel 1D line lattices of black (degree-3) nodes are stacked along the *z* direction. The layers alternate between the line lattices pointing in the *x* and *y* directions. Neighboring layers are straddled by a layer of red (degree-2) nodes. Along each line lattice of black nodes, the blue bonds alternate between the +*z* and −*z* directions. The adjective “modified” we use in the name of this lattice refers to the fact that in the standard (10,3)-b lattice, the red nodes are not present, i.e., the adjacent (*x*, *y*) planes of parallel lattices in alternating directions are directly connected via bonds

Equipped with this new formalism, we construct a 3D logical lattice G_2_—a modified (10,3)-b lattice—shown in Fig. 5. In this case, G_3_, the lattice connecting the red nodes of G_2_ if all the fusions were to succeed, is the 3D diamond lattice. The bond-percolation threshold of the modified (10,3)-b lattice, and hence the threshold on fusion success probability that would ensure ${\mathrm{G}}_1^\prime$ to be long-range connected, is *p*_c_(G_2_) ≈ 0.627. Therefore, ${\lambda }_{\mathrm{c}}^{(3)} \le 0.627$, which gives a better (lower) upper bound than the example we examined above.

Let us see how and why G_3_ for this example is in the diamond lattice. Let us assume that all the fusion attempts succeed, so that the black nodes of G_2_ in Fig. 5 are gone. Let us pick any red node and follow the blue-dashed edge up to the neighboring black node. The black node has two neighboring black nodes, each of which is connected to a red node via blue-dashed edges. Once the photons within the black nodes have disappeared after the fusions, the red node we started with will inherit these two red nodes as its immediate neighbors. Similarly, if we followed the blue-dashed edge down from the original red node we picked, we would find two other red-node neighbors of that red node after the fusions are done. So, if all the fusion attempts were to succeed, we will get a regular 3D lattice of red nodes, where each node has degree 4. This is the diamond lattice.

The authors of ref. ^18^ did exactly the above, attempting to create a percolated instance of the 3D diamond lattice as a long-range-connected photonic lattice. But our reinterpretation of their (nonstandard) percolation threshold as a standard bond-percolation threshold of the modified (10,3)-b lattice not only provides a natural way to account for failure modes of fusion attempts, but allows one to exploit existing literature on efficient Monte Carlo methods (viz., the Newman–Ziff method^26^) to calculate sharper percolation thresholds. More importantly, this interpretation also opens the door for new constructions and improved thresholds, which we describe next.

### Improved thresholds

Leveraging this new insight, we embark upon a progression of improved achievability thresholds for *n* = 3, using higher-dimensional generalizations of the modified (10,3)-b lattice.

**Fig. 6:**
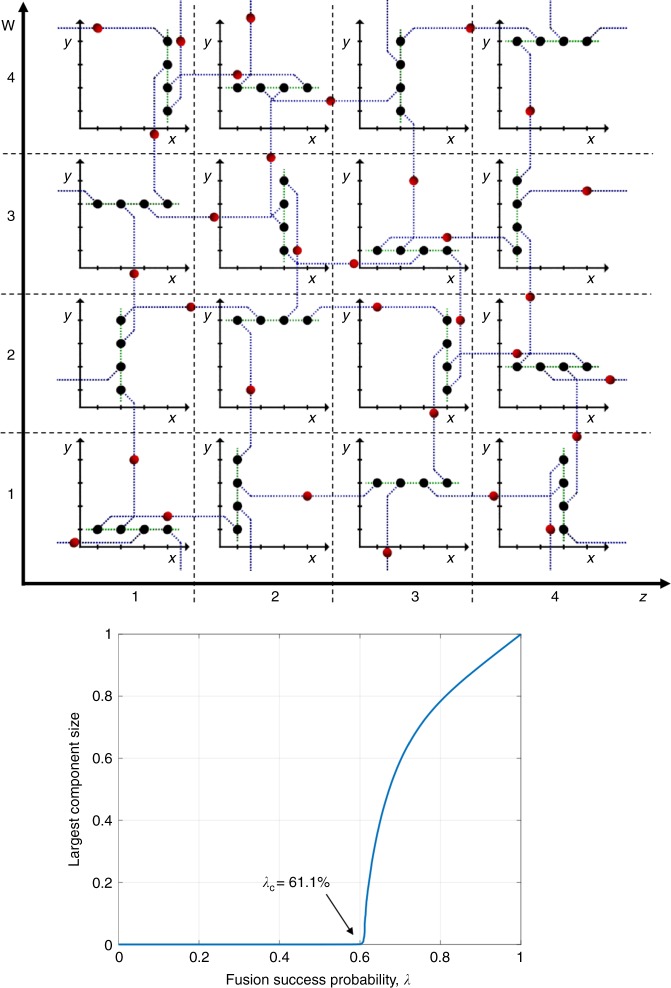
Schematic of the 4D extension of the (10,3)-b lattice. The inner plots with *x* and *y* axes represent projections of the lattice on the (*x*, *y*) plane at the *z* and *w* values shown on the outer axes

First, we consider a 4D extension of the (10,3)-b lattice (see Fig. 6). It consists of a doubly infinite stack of (*x*, *y*)-plane layers—of parallel 1D line lattices of black (degree-3) nodes—stacked along the *z* and *w* directions, respectively. Of the three neighboring bonds of a black node, two (green) bonds—connecting to neighboring black nodes in the line lattice to which it belongs—are in the (*x*, *y*) plane, whereas one (blue) bond—connecting to a red node, which in turns connects via another blue bond to a black node in a neighboring (*x*, *y*)-plane layer—points in either the *z* direction or in the *w* direction. Along each line lattice of black nodes, the blue bonds alternate between directions +*z*, +*w*, −*z*, −*w*, …, and so on. The graph has a period of four in each of the *x*, *y*, *z*, and *w* dimensions. One period of the lattice is depicted in Fig. 6. The inner axes represent an (*x*, *y*) plane at a given value of *z* and *w*. This construction results in longer loops compared with the 3D case discussed above, while retaining the 3D graph’s coordination number (average node degree), which in turn lowers the bond-percolation threshold. We find, using a Newman–Ziff simulation performed on a 4D-modified (10,3)-b lattice of size *N* ~ 10^7^ nodes, that

5 $${\lambda }_{\mathrm{c}}^{(3)} \le 0.611.$$

**Fig. 7:**
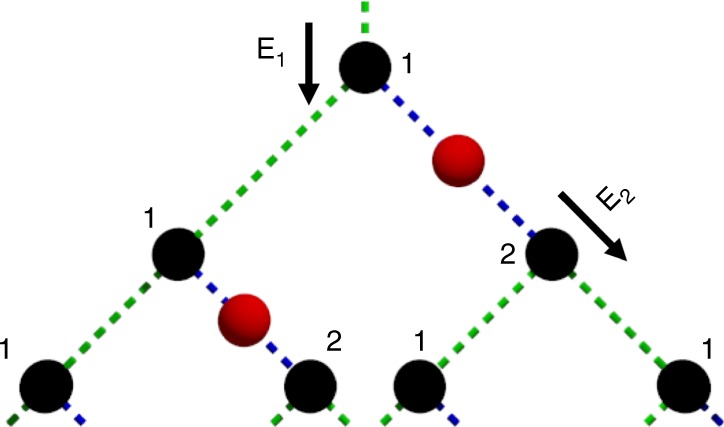
Schematic of the ∞-D extension of the (10,3)-b lattice. This lattice when used as the logical graph with node colors as shown yields *λ*_c_ ≈ 0.5898. Percolation threshold was evaluated analytically

Next, we consider an infinite-dimensional modified (10,3)-b lattice, which becomes locally tree-like, as shown in Fig. 7. However, note that for any finite dimension of this family of modified (10,3)-b lattices, no matter how large the dimension is, it is not a tree, and a percolated subgraph of it will give us a renormalizable universal cluster. Similar to the 3D- and 4D-modified (10,3)-b lattices, each black node has two green bonds and one blue bond (which in turn leads to a black node via a red node and another blue bond). We denote the expected number of children of a node when approached via a green bond as *E*_1_ and the expected number of children of a node when approached via a blue bond as *E*_2_. When counting the number of children of a node, we only count red nodes since they are the only nodes with unmeasured qubits. Counting children from the top of Fig. 7, each black node is labeled as 1 or 2 depending on the bond from which it is approached. Counting children at the points labeled *E*_1_ and *E*_2_ yields the equations *E*_1_ = *λE*_1_ + *λ* + *λ*^2^*E*_2_ and *E*_2_ = 2*λE*_1_, where *λ* is the bond probability. For percolation, *E*_1_ → ∞ and solving the equations with this condition, we find that ${\lambda }_{\mathrm{c}} + 2{\lambda }_{\mathrm{c}}^3 = 1$, which leads to *λ*_c_ ≈ 0.5898. Therefore,

6 $${\lambda }_{\mathrm{c}}^{(3)} \le 0.5898,$$

thereby bringing the best-known achievable threshold with three-photon clusters as the initial resource closer to our general lower bound applied to *n* = 3, ${\lambda }_{\mathrm{c}}^{(3)} \ge 0.5$.

### Universal cluster creation on a 2D planar lattice

**Fig. 8:**
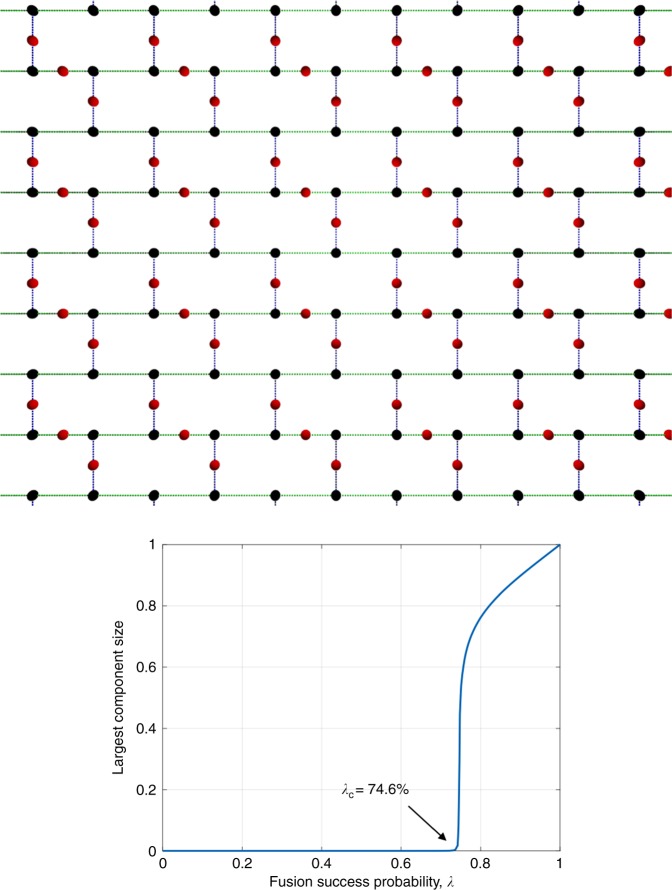
Bond percolation on the modified 2D brickwork lattice. This 2D lattice has a bond-percolation threshold of 0.746, which is less than the fusion success probability achievable with single-photon boosting. This shows that single-photon-boosted linear optical fusion and three-photon GHZ- state clusters are sufficient to generate a universal lattice ballistically by fusing the clusters on a two-dimensional lattice

We found a 2D logical lattice, a modified brickwork (see Fig. 8), for which *λ*_c_ = 0.746. This gives a bound ${\lambda }_c^{(3)} \le 0.746$, which is looser, compared with the ones we obtained from the constructions in the previous section. However, since 0.746 < 25/32, this result shows that single-photon-boosted linear optical fusion and three-photon GHZ-state clusters are sufficient to generate a universal lattice ballistically by fusing the clusters on a two-dimensional lattice. Fusion on a 2D lattice is significantly simpler, compared with 3D (or higher-dimensional) lattices from an experimental standpoint, since 2D programmable linear optics is a fast-maturing technology. Whether or not the above is possible was posed as a challenging problem by Rudolph and colleagues in refs. ^18,19^.

### Effects of photon loss on the thresholds

**Fig. 9:**
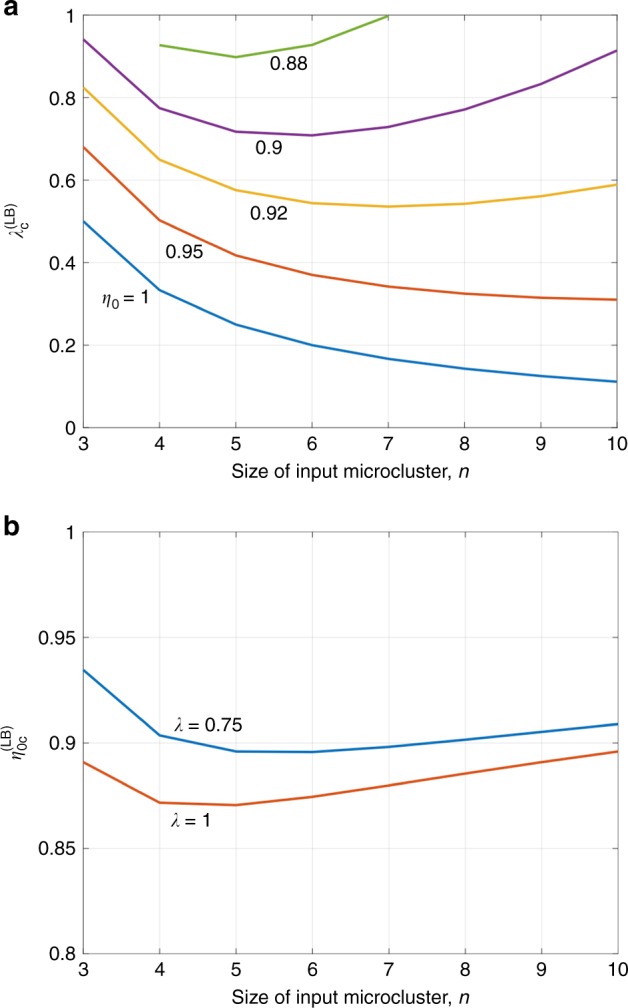
Effect of photon loss on thresholds. **a** A loss-dependent lower bound ${\lambda }_{\mathrm{c}}^{({\mathrm{LB}})}$ on the critical fusion probability *λ*_c_ as a function of the input microcluster size *n* for different values of *η*_0_; (**b**) a loss-dependent lower bound $\eta _{0{\mathrm{c}}}^{({\mathrm{LB}})}$ on the critical loss parameter *η*_0_ as a function of *n* for different values of fusion success probability *λ*

We prove an extension of our lower bound on ${\lambda }_{\mathrm{c}}^{(n)}$. We use a loss model inspired by proposals to produce photonic microclusters using quantum emitters^21,27,28^, which have been recently realized experimentally^22^. In these proposals, since each node of the microcluster is added sequentially, we assume that the total transmissivity of the photon in its lifetime is of the form $\eta = \eta _0^n$ (the loss is 1−*η*) where *n* is the size of the microcluster and *η*_0_ represents the transmissivity in one step. Hence, the total loss seen by the photon increases with the size of the microcluster. The import fidelity of the clusters generated due to unheralded photon losses and other forms of errors (e.g., mode mismatch, detector dark clicks) have not been considered. To obtain a lower bound, we only look at loss in the photons undergoing fusion and observe that a fusion gate only succeeds if both input photons are detected. Furthermore, for our bound, we treat loss as the removal of the corresponding node from the cluster state or a measurement in the *Z* basis. In general, loss of a qubit is not equivalent to a *Z*-basis measurement. Loss results in tracing out the qubit, but treating it as a *Z*-basis measurement leads to a lower value of *λ*_c_ (*Z*-basis measurement gives us more information than tracing out over the *Z* basis) and is sufficient for a lower bound on ${\lambda }_{\mathrm{c}}^{(n)}$. Hence, if ${\lambda }\eta _0^{2n} < 1/(n - 1)$, there cannot be a giant-connected component in the cluster, and we obtain the loss-dependent lower bound of ${\lambda }_{\mathrm{c}} \ge 1/[(n - 1)\eta _0^{2n}] \equiv {\lambda }_{\mathrm{c}}^{({\mathrm{LB}})}$. Figure 9 plots ${\lambda }_{\mathrm{c}}^{({\mathrm{LB}})}$ as a function of *n* for different values of *η*_0_ and we find that there is an optimum value of *n* for any *η*_0_ < 1, which gives the lowest ${\lambda }_{\mathrm{c}}^{({\mathrm{LB}})}$. In other words, there is an optimum size of the starting microcluster, for which the required fusion probability is minimized.

Conversely, for a given fusion success probability *λ*, there exists a threshold *η*_0c_, s.t., if *η*_0_ < *η*_0c_, the post-fusion cluster cannot be percolated. We thus have a lower bound $\eta _{0{\mathrm{c}}} \ge \eta _{0{\mathrm{c}}}^{{\mathrm{(LB)}}}$, where $\eta _{0{\mathrm{c}}}^{{\mathrm{(LB)}}} = \left[ {1/{\lambda }(n - 1)} \right]^{1/(2n)}$. In Fig. 9b, we plot $\eta _{0{\mathrm{c}}}^{({\mathrm{LB}})}$ for different values of *n*, for *λ* = 0.75 and *λ* = 1. There is an optimum value of *n* which gives the best lower bound on loss tolerance, e.g., for *λ* = 0.75, six-photon microclusters give the lowest bound of $\eta _{0{\mathrm{c}}}^{{\mathrm{(LB)}}} = 0.8957$ which corresponds to a loss (1 − *η*) of 48.36% seen by each photon. Furthermore, we find that going from *λ* = 0.75, which is attainable using four single- ancilla photons and (lossless) linear optics^8^ to deterministic fusion (*λ* = 1), $\eta _{0{\mathrm{c}}}^{({\mathrm{LB}})}$ only decreases slightly, i.e., the equivalent per-photon loss threshold increases from 89.6 to 87.1%. Hence, when losses are accounted for in ballistic cluster state creation, the advantage in having a fully deterministic fusion may be relatively small. It is important to note, however, that the numbers presented here are only lower bounds on *η*_0c_ and *λ*_0c_, and may not be tight. For example, our results show that any ballistic cluster creation process that starts with six-photon microclusters and uses destructive fusion with *λ* = 0.75 cannot tolerate more than 48.36% loss. However, these results do not prove that 48.36% loss is sufficient for ballistic cluster creation.

## Discussion

Despite recent progress in LOQC making it resurface as a strong contender to scalable quantum computing, many questions remain, whose answers will be crucial to its eventual success. A major challenge, as expounded in ref. ^23^ as well, is an experimental scheme to build an on-demand source of high-fidelity small entangled cluster states, e.g., three-photon GHZ states. The other big experimental challenge is to realize high-quality quantum interference of the photon clusters using a linear optical interferometer that minimizes in-line losses and mode mismatch.

A few immediate problems we leave open in this paper are as follows: (a) proving our conjecture on the achievability of ${\lambda }_{\mathrm{c}}^{(n)} = 1/(n - 1) + \varepsilon$; and (b) for a given *n*, finding the construction that gives the smallest overhead (i.e., edge length of an optimally chosen renormalized universal lattice), while not limiting only to those linear–optical unitaries *U* that can be assembled with two-qubit fusion gates (i.e., allow for higher-dimensional linear–optical projective operations to potentially fuse more than two clusters at once).

On the theoretical front, some of the most important open problems are as follows: (1) extending this work to include mode mismatch within the linear optic fusion circuits; (2) finding the minimum size clusters and the most resource-efficient schemes for ballistic cluster creation that are immune to unheralded losses.

In this paper, we only considered losses in photons undergoing fusion. This was sufficient for obtaining bounds on ${\lambda }_{\mathrm{c}}^{(n)}$ and *η*_0c_. However, any losses in photons that do not undergo fusion are unheralded, i.e., we do not know which photons were lost. The strategy of renormalizing by finding criss-crossing highways and choosing the intersection of these highways to be logical qubits, which gives us a perfect universal cluster state in the lossless case, fails in the presence of such unheralded loss. This is because even if we have a percolated cluster, we need to know the obtained graph state exactly in order to compute the highways. In order to correct for these unheralded photon losses (and other forms of qubit errors), an additional layer of error correction is needed^29–33^. Let us consider five-photon GHZ states as a starting point, which correspond to five node clusters in the “star” configuration, and place them at the nodes of a Raussendorf lattice (which has degree-4 nodes). The central photon of the star is placed on a node of the Raussendorf lattice and the four leaf nodes of the star are placed along the four neighboring bonds. We now attempt two-qubit fusion on each bond, attempting to construct a bond-percolated instance of the Raussendorf lattice following the recipe in ref. ^15^. If each fusion attempt succeeds with probability λ, we obtain a subgraph of the Raussendorf lattice where each bond is present independently with probability λ. Recent work has shown that a subgraph of the Raussendorf lattice with 0.145 or a smaller fraction of missing bonds^33^ (or with 0.249 or a smaller fraction of missing qubit sites^30^) can be used for universal quantum computation while correcting for unheralded qubit errors. The amount of unheralded error on each physical qubit that can be corrected using such a thinned Rausendorf lattice depends upon how far below 0.145 the actual fraction of missing bonds is. While unheralded photon losses can be accounted for with such a construction starting with five-photon clusters as described above, it requires a fusion probability exceeding 0.85, which is greater than the best known linear optic Bell measurements boosted with single photons or squeezing. Future work should look at ways of reducing the required fusion probability to within 0.78—which is achievable with single-photon boosting—by using more efficient constructions and with smaller clusters as the starting point, and one should investigate experimentally realizable methods that improve upon the achievable fusion success probability beyond 0.78. It will also be important to extend the error-correction method to account for mode-mismatch errors in the linear optic mixing in the fusion gates, as well as detector excess noise, such as dark clicks.

### Code availability

All the numerical data presented in this paper are the results of C simulations conducted by M.P. The code used to generate thiese data will be made available to the interested reader upon reasonable request.

## Supplementary information

Supplementary Information

Peer Review File

## Data Availability

The data sets generated during and or analyzed during the current study are available from the corresponding author on reasonable request.

## References

[1] Reck M, Zeilinger A (1994). Experimental realization of any discrete unitary operator. Phys. Rev. Lett..

[2] Carolan J (2015). Universal linear optics. Science.

[3] Harris NC (2017). Quantum transport simulations in a programmable nanophotonic processor. Nat. Photonics.

[4] Calsamiglia J, Lütkenhaus N (2001). Maximum efficiency of a linear-optical Bell-state analyzer. Appl. Phys. B.

[5] Michler M, Mattle K, Weinfurter H, Zeilinger A (1996). Interferometric Bell-state analysis. Phys. Rev. A..

[6] Grice WP (2011). Arbitrarily complete Bell-state measurement using only linear optical elements. Phys. Rev. A..

[7] Zaidi HA, van Loock P (2013). Beating the one-half limit of ancilla-free linear optics Bell measurements. Phys. Rev. Lett..

[8] Ewert F, van Loock P (2014). 3/4-efficient Bell measurement with passive linear optics and unentangled ancillae. Phys. Rev. Lett..

[9] Pan Jw, Zeilinger A (1998). Greenberger-Horne-Zeilinger-state analyzer. Phys. Rev. A..

[10] Aaronson, S. & Arkhipov, A. The computational complexity of linear optics. *Proceedings of the 43rd annual ACM symposium on Theory of computing—STOC ‘11*. 333–342 (ACM Press: New York, San Jose, California, USA, June 06–08, 2011. 10.1145/1993636.1993682.

[11] Knill E, Laflamme R, Milburn GJ (2001). A scheme for efficient quantum computation with linear optics. Nature.

[12] Raussendorf R, Briegel HJ (2001). A one-way quantum computer. Phys. Rev. Lett..

[13] Browne DE, Rudolph T (2005). Resource-efficient linear optical quantum computation. Phys. Rev. Lett..

[14] Kok P, Nemoto K, Ralph TC, Dowling JP, Milburn GJ (2007). Linear optical quantum computing with photonic qubits. Rev. Mod. Phys..

[15] Kieling, K., Rudolph, T. & Eisert, J. Percolation, renormalization, and quantum computing with nondeterministic gates. *Phys. Rev. Lett.* **99**, 130501 (2007). 10.1103/PhysRevLett.99.130501 17930565

[16] Browne DE (2008). Phase transition of computational power in the resource states for one-way quantum computation. New J. Phys..

[17] Grimmett G (1999). Percolation, vol. 321 of Grundlehren der mathematischen Wissenschaften.

[18] Gimeno-Segovia M, Shadbolt P, Browne DE, Rudolph T (2015). From three-photon Greenberger-Horne-Zeilinger states to ballistic universal quantum computation. Phys. Rev. Lett..

[19] Zaidi HA, Dawson C, Van Loock P, Rudolph T (2015). Near-deterministic creation of universal cluster states with probabilistic Bell measurements and three-qubit resource states. Phys. Rev. A - At., Mol., Opt. Phys..

[20] Kieling K, Eisert J (2009). Percolation in quantum computation and communication. Lect. Notes Phys..

[21] Rao DDB, Yang S, Wrachtrup J (2015). Generation of entangled photon strings using NV centers in diamond. Phys. Rev. B - Condens. Matter Mater. Phys..

[22] Schwartz I (2016). Deterministic generation of a cluster state of entangled photons. Science.

[23] Rudolph T (2017). Why I am optimistic about the silicon-photonic route to quantum computing. APL Photonics.

[24] Morley-Short S (2018). Physical-depth architectural requirements for generating universal photonic cluster states. Quantum Sci. Technol..

[25] Tran J, Yoo T, Stahlheber S, Small A (2013). Percolation thresholds on three-dimensional lattices with three nearest neighbors. J. Stat. Mech.: Theory Exp..

[26] Newman MEJ, Ziff RM (2001). Fast Monte Carlo algorithm for site or bond percolation. Phys. Rev. E - Stat., Nonlinear, Soft Matter Phys..

[27] Lindner NH, Rudolph T (2009). Proposal for pulsed On-demand sources of photonic cluster state strings. Phys. Rev. Lett..

[28] Economou SE, Lindner N, Rudolph T (2010). Optically generated 2-dimensional photonic cluster state from coupled quantum dots. Phys. Rev. Lett..

[29] Varnava M, Browne D, Rudolph T (2006). Loss tolerance in one-way quantum computation via counterfactual error correction. Phys. Rev. Lett..

[30] Barrett SD, Stace TM (2010). Fault tolerant quantum computation with very high threshold for loss errors. Phys. Rev. Lett..

[31] Michael MH (2016). New class of quantum error-correcting codes for a bosonic mode. Phys. Rev. X.

[32] Herr D, Paler A, Devitt SJ, Nori F (2018). A local and scalable lattice renormalization method for ballistic quantum computation. npj Quantum Inf..

[33] Auger JM, Anwar H, Gimeno-Segovia M, Stace TM, Browne DE (2018). Fault-tolerant quantum computation with nondeterministic entangling gates. Phys. Rev. A..

