# Supplementary material for: Percolation thresholds for photonic quantum computing
Source: Nat Commun. 2019 Mar 6;10:1070. doi: 10.1038/s41467-019-08948-x (PMC6403388; doi:10.1038/s41467-019-08948-x)
Supplement: Supplementary file 1 — Supplementary Information [file 41467_2019_8948_MOESM1_ESM.pdf]

Reviewers' comments:

Reviewer #1 (Remarks to the Author):

This paper applies ideas from percolation theory to develop bounds on the fusion statistics for qubit fusion. The authors use state-of-the art methods in percolation theory to analyze lattices not previously analyzed, in two, three, four and infinite (Bethe lattice) dimensions. The results should be useful for future design of qubit systems. I highly recommend publication.

Reviewer #2 (Remarks to the Author):

SUMMARY

As the title indicates, the authors study percolation thresholds for photonic quantum computing. This work provides important general bounds for successful generation of photonic universal resource states for quantum computing, answers important open questions previously posed as conjectures, and provides an elegant graph-theoretic framework for studying the creation of general graph states from microclusters and probabilistic fusion operations (2-mode and beyond).

REVIEW

This is an important, significant, well-written contribution to the literature regarding percolation-based approaches to photonic quantum computing. For all of the reasons given in my summary above, I recommend publication after the following are properly addressed:

1. I question the wording immediately below Eq. (6) -- i.e., the claim that the authors have "almost clos[ed] the gap" to the general lower bound for n=3. After all, 0.5898 is closer to 0.611 than it is to 0.5, so there is still a large (proportional) distance to go to close this gap. Perhaps the authors could soften this language.

2. At the beginning of Section II.E, the authors should double check the direction of the inequality on \lambda_c^{(3)} \geq 0.746. Should it perhaps be \leq instead? (That is, isn't it possible that there could be a lower value of \lambda_c^{(3)} obtained from an even-more-clever choice of graph?)

3. In Section II.F, it isn't quite accurate to say that "[p]hoton loss is *equivalent* to measurement by the environment in the Z basis, with an unknown measurement result..." (emphasis mine). The operation described is merely dephasing in Z. Photon loss would be equivalent to complete depolarisation, with a heralding cue or not depending on whether the loss is heralded. The reason this analogy works for the purposes to which the authors are using are putting it is that (a) the state being measured is unbiased in the Z basis, which means that dephasing results in a maximally mixed state anyway, and (b) the noise model can be weakened as much as one likes because it is being used to prove the impossibility of creating a giant connected component under certain conditions. Nevertheless, the authors should correct this language -- lest an unfortunate graduate student think this is how loss can be modelled in all cases.

4. Section I of the Supplementary Material would really benefit from a few diagrams. I was able to follow the proof, but an illustration or two would help. So would a brief outline of the method before diving into the mathematics. As such... (next item)

5. Also in Section I of the supplementary Material, I would recommend adding a sentence or two at the beginning giving some intuition for how the F_n, L, and S are going to be used. This becomes clear as the proof progresses, but some foreshadowing of their roles as they are

introduced would really help the reader.

6. The final step in proving (the first-named) Lemma 3 involves a claim about bond percolation on a graph with maximum degree n. The authors should add a rigorous proof -- or a citation to such a proof -- justifying the particular claim being used (regarding o(N) scaling).

7. The end of Section I of the Supplementary Material states that "[t]he three lemmas can be proved with the same reasoning...", but the "proof" of (the second-named) Lemma 3 (which should receive a new, unique number, as requested below) should be more specific since there is a new parameter m and a new claim being made about what happens when \lambda < 1/(n-1)(m-1). It's plausible that (this second instance of) Lemma 3 might be true, but the authors should add an explicit proof or a citation to such a proof.

8. On page 5 of the Supplementary Material, it is argued that "it is simple to argue that there are O(N) red nodes in the GCC." The authors should state this "simple" argument explicitly.

9. Section III.A of the Supplementary Material refers only once to "the Bethe lattice above". Using this terminology in isolation is confusing. If the authors wish to retain it, then they should state exactly where "above" they are referring, and at that "above" location, they should label the lattice as a "Bethe lattice" (I'm assuming it's the one in Fig. 3).

10. At the very end of page 5 of the Supplementary Material, the authors refer to "yellow dashed lines". The dashed lines in Fig. 5 don't look yellow to me. This may just be a problem with my printer, but if they're not actually yellow, the authors should correct this.

11. In the first bit of text on page 6 of the Supplementary Material, the phrase "Z measurement followed by a Hadamard gate" should probably be reversed to become "Hadamard gate followed by a Z measurement" since it makes no sense to do a gate on a qubit that has been measured -- and because the effect of an X measurement (H followed by Z-meas.) is what is being described.

12. The authors should ensure that the abbreviations 'GCC' and 'LCC' have been properly defined before use in all sections of the main text and also separately in the Supplementary Material.

13. Typos
* (main) page 5, right column, near the top: "black bonds" should probably be "black nodes" (?)
* (main) page 6, left column, near middle: "it's lifetime" should be "its lifetime"
* (main) page 7, left column, near top: "high-Fidelity" should be "high-fidelity"
* (supplement) page 1, left column, near top: "if there are a path" should be "if there is a path"
* (supplement) page 2, left column, about 1/3 of the way down: within "L_{0k} = \{ v_{al}, v_{bl} \}", the subscript {bl} should probably be {bm} (I think).
* (supplement) page 2, left column, final paragraph: "if \{ v_a, v_b \} if" should be "if \{ v_a, v_b \} is"
* (supplement) page 2, right column, near top: The sentence "From Lemma 3..." is grammatically incorrect.
* (supplement) page 2, right column, about 2/3 of the way down: "every vertex is" should probably be "every vertex" (?)

14. Lemmas and Theorems in the Supplementary Material: The authors should use the 'amsthm' LaTeX package and its included {theorem}, {proof}, etc. environments. This ensures that lemmas and theorems are numbered uniquely throughout the document and that they and their proofs are formatted correctly (including ending proofs with a \qed symbol).

Once these changes are properly addressed, I strongly recommend publication.

Reviewer #3 (Remarks to the Author):

In the manuscript, titled “Percolation thresholds for photonic quantum computing”, authors studied linear optical quantum computing using the ballistic strategy and presented some interesting results.

Using linear optics to entangle photons is one of the most promising way of realising quantum computing. Preparing scalable cluster states is the critical step in linear optical quantum computing. Ballistic protocols are attractive protocols for preparing scalable cluster states. Ballistic protocols are not well studied as the alternative family of protocols, KLM and its variants. It is still an open question whether the performance of ballistic protocols can be superior to KLM family protocols. However, we know that KLM family protocols require a huge number of linear optical instruments [e.g. see PRX 5, 041007 (2015)]. It may turn out to be the case that the optimal protocol combines two strategies. General conclusions about the ballistic strategy are important.

The manuscript presented two general conclusions, which are the first two of four results as summarised by authors. However, these two conclusions are not sufficiently justified. Therefore, I do not recommend the publication of the manuscript.

About the first general conclusion:

The first general conclusion (main result 1) has two parts. The first part is a lower bound of the success probability threshold. If the success probability is higher than the threshold, scalable cluster states can be prepared using a ballistic protocol. The second part is a conjecture that the bound is tight. If the conjecture is true, a success probability higher than the lower bound is enough for preparing scalable cluster states.

All evidences supporting the second part are given in the appendix. I do not find that these evidences can support a reasonable conjecture accounted as a main result of a paper. I recommend authors to either move the second part from the first main result or provide more evidences. As a main result, authors should at least explain in the main text why they conjecture that the second part is true.

About the second general conclusion:

The second general conclusion may be more useful. Authors find that the success probability threshold of a ballistic protocol is the standard bound percolation threshold on a lattice. This result can help us in looking for the optimal protocol of linear optical quantum computing.

The second general conclusion is sound. However, why the bound percolation threshold of the fusion-operation lattice is the success probability threshold is not proved or explicitly explained. Actually, it may not be difficult to prove their conclusion. I recomend authors to explicitly give the proof rather than hand-waving.

Other comments and questions:

1. It is costly to generate n-photon states starting from single photons. If this stage is considered, is it still that case that a larger n has advantage, because the lower bound decreases with n? Generating n-photon states using emitters is also discussed in the manuscript. How good the emitters should be in terms of the loss rate and fidelity?

2. "Seeing this requires a bit of imagination, and is not shown in Fig. 4." If it is not shown in the figure, authors should explain it a bit rather than rely on reader's imagination. On the diamond lattice, only red nodes are left, and each red node is connected to a neighbour red node with

distance 3 in Fig. 4.

3. Usually, eta is used to denote efficiency rather than loss. Efficiency = 1 - Loss. Authors should clarify what eta means in the manuscript. For example, in the sentence "the total loss seen by a photon in it’s lifetime is of the form eta = ...", eta is efficiency in the equation but it is called total loss.
This question leads to another question. "a loss of 48.36% seen by each photon." Here, is 48.36% efficiency or loss? If it is efficiency and lower than 50%, does it mean that two scalable cluster states can be prepared rather than one using the protocol?

Response to Referee's comments on Manuscript number NCOMMS-18-04990A, "Percolation thresholds for photonic quantum computing"

M. Pant, D. Towsley, D. Englund and S. Guha

September 30, 2018

Reviewers' comments:

Reviewer #1 (Remarks to the Author):

This paper applies ideas from percolation theory to develop bounds on the fusion statistics for qubit fusion. The authors use state-of-the art methods in percolation theory to analyze lattices not previously analyzed, in two, three, four and infinite (Bethe lattice) dimensions. The results should be useful for future design of qubit systems. I highly recommend publication.

We thank reviewer 1 for taking the time to read our paper and the highly positive review.

Reviewer #2 (Remarks to the Author):

SUMMARY

As the title indicates, the authors study percolation thresholds for photonic quantum computing. This work provides important general bounds for successful generation of photonic universal resource states for quantum computing, answers important open questions previously posed as conjectures, and provides an elegant graph-theoretic framework for studying the creation of general graph states from microclusters and probabilistic fusion operations (2-mode and beyond).

We thank review 2 for taking the time to read our manuscript, and the constructive feedback.

REVIEW

This is an important, significant, well-written contribution to the literature regarding percolation-based approaches to photonic quantum computing. For all of the reasons given in my summary above, I recommend publication after the following are properly addressed:

We thank reviewer 2 for recommending publication of our manuscript upon successfully addressing the comments below. Please see our response in line with the feedback below, and description of changes we have made to the manuscript, as necessary to address those.

1. I question the wording immediately below Eq. (6) -- i.e., the claim that the authors have "almost clos[ed] the gap" to the general lower bound for n=3. After all, 0.5898 is closer to 0.611 than it is to 0.5, so there is still a large (proportional) distance to go to close this gap. Perhaps the authors could soften this language.

The 0.611 achievability threshold in Eq. (5) is one of our (new) results in this paper. This threshold corresponds to our new graphical method applied to the 4-dimensional extension of the generalization of the (10,3)-b lattice family we propose in this paper. Before this paper, the best-known threshold was ~0.625 (diamond lattice from Rudolph and colleagues). We changed the language to:

"thereby bringing the best-known achievable threshold with 3-photon clusters as the initial resource closer to our general lower bound applied to $n=3$, $\lambda_c^{(3)} \ge 0.5$."

2. At the beginning of Section II.E, the authors should double check the direction of the inequality on \lambda_c^{(3)} \geq 0.746. Should it perhaps be \leq instead? (That is, isn't it possible that there could be a lower value of \lambda_c^{(3)} obtained from an even-more-clever choice of graph?)

We apologize for the confusion wording in that paragraph. Please see below the modified wording of that paragraph:

We found a 2D logical lattice, a modified brickwork (see Fig.~\ref{fig:2Dmodifiedbrickwork}), for which $\lambda_c = 0.746$. This gives a bound $\lambda_c^{(3)} \leq 0.746$, which is looser compared to the ones we obtained from the constructions in the previous section. However, since $0.746 < 25/32$, this result shows that single-photon-boosted linear optical fusion and 3-photon GHZ state clusters are sufficient to generate a universal lattice ballistically by fusing the clusters on a {\em two-dimensional} lattice. Fusion on a 2D lattice is significantly simpler compared to 3D (or higher dimensional) lattices from an experimental standpoint, since 2D programmable linear optics is a fast-maturing technology. Whether or not the above is possible was posed as a challenge problem by Rudolph and colleagues in~\cite{2015.PRL.Gimeno-Segovia-Rudolph.3GHZtoBallisticQC,2015.PRA.Zaidi-Rudolph.BallisticLOQC}.

*3. In Section II.F, it isn't quite accurate to say that "[p]hoton loss is *equivalent* to measurement by the environment in the Z basis, with an unknown measurement result..." (emphasis mine). The operation described is merely dephasing in Z. Photon loss would be equivalent to complete depolarisation, with a heralding cue or not depending on whether the loss is heralded. The reason this analogy works for the purposes to which the authors are using are putting it is that (a) the state being measured is unbiased in the Z basis, which means that dephasing results in a maximally mixed state anyway, and (b) the noise model can be weakened as much as one likes because it is being used to prove the impossibility of creating a giant connected component under certain conditions. Nevertheless, the authors should correct this language -- lest an unfortunate graduate student think this is how loss can be modelled in all cases.*

We have clarified this in the revised draft. Please see below the modified wording:

Furthermore, for our bound, we treat loss as the removal of the corresponding node from the cluster state or a measurement in the Z basis. In general, loss of a qubit is not equivalent to a Z basis measurement. Loss results in tracing out the qubit, but treating it as a Z basis measurement leads to a lower value of λ_c (Z basis measurement gives us more information than tracing out over the Z basis) and is sufficient for a lower bound on $\lambda_c^{(n)}$.

4. Section I of the Supplementary Material would really benefit from a few diagrams. I was able to follow the proof, but an illustration or two would help. So would a brief outline of the method before diving into the mathematics. As such... (next item)

5. Also in Section I of the supplementary Material, I would recommend adding a sentence or two at the beginning giving some intuition for how the F_n, L, and S are going to be used. This becomes clear as the proof progresses, but some foreshadowing of their roles as they are introduced would really help the reader.

We have added a proof outline to the beginning of Section I which now foreshadows how the roles of the different pieces of the proof and also provide intuition to the reader. Furthermore, we have added Fig. 1 to the supplementary material which depicts the mapping between the different operations used in the proof to guide the reader.

6. The final step in proving (the first-named) Lemma 3 involves a claim about bond percolation on a graph with maximum degree n. The authors should add a rigorous proof -- or a citation to such a proof -- justifying the particular claim being used (regarding o(N) scaling).

We have added citations which contain the proof (Ref. 1, 2 in the supplementary information).

7. The end of Section I of the Supplementary Material states that "[t]he three lemmas can be proved with the same reasoning...", but the "proof" of (the second-named) Lemma 3 (which should receive a new, unique number, as requested below) should be more specific since there is a new parameter m and a new claim being made about what happens when \lambda < 1/(n-1)(m-1). It's plausible that (this second instance of) Lemma 3 might be true, but the authors should add an explicit proof or a citation to such a proof.

We have changed the numbering of the Lemmas used in the generalization to the m node fusions to 4,5 and 6. Furthermore, these Lemmas now have their own separate proofs in the revised draft that can be read independently. We have also added Fig. 2 to the supplementary material to aid the explanation of Lemma 6.

8. On page 5 of the Supplementary Material, it is argued that "it is simple to argue that there are O(N) red nodes in the GCC." The authors should state this "simple" argument explicitly.

We have modified the following paragraphs leading up to the argument, to make this explicit.

~~
Let us say there are N unmeasured photons in the lattice in Fig.~\ref{fig:Keilingtonow}(b). It is clear from the figure that there are $8N$ photons that get measured, making it a total of $9N$ photons. The logical graph in Fig.~\ref{fig:Keilingtonow}(e) has $3N$ nodes, where each node represents a 3-photon (GHZ) cluster.

Each bond of the logical graph G represents a fusion attempt. All the photons within the black nodes disappear after the fusion attempts have been made but help provide long-range connections, if more than λ_c fraction of the bonds (fusion attempts) were successful. Only the red nodes, which in the example of Fig.~\ref{fig:Keilingtonow}(e) contain a single photon each after the fusion attempts have been made, remain as part of the resulting connected component(s) comprised of the unmeasured photons in the red nodes.

If the individual fusions succeed with probability, $\lambda > \lambda_c$, the bond percolation threshold of G, with high probability, the largest connected component (LCC) will comprise of $O(N)$ unmeasured photons, also known as giant connected component (GCC). The constant in this $O(N)$ is given by the y-axis in the plot in Fig.~\ref{fig:Keilingtonow}(e). This GCC is subsequently renormalized into a perfect square grid of $O(N)$ nodes, which is used for cluster-model quantum computing.

These $O(N)$ nodes have both red nodes and black nodes. However, since every black node has a red node at a constant distance (one bond away) from it, the probability of a red node being connected to the nearest black node is a constant. Therefore, the presence of $O(N)$ black nodes in the GCC implies the presence of $O(N)$ red nodes in the GCC, and hence, there must be $O(N)$ red nodes in the GCC whenever there are $O(N)$ nodes in the GCC.

~~

9. Section III.A of the Supplementary Material refers only once to "the Bethe lattice above". Using this terminology in isolation is confusing. If the authors wish to retain it, then they should state exactly where "above" they are referring, and at that "above" location, they should label the lattice as a "Bethe lattice" (I'm assuming it's the one in Fig. 3).

Referring to the graph as the Bethe lattice is not necessary and the reference has just been changed to ”the lattice above”

10. At the very end of page 5 of the Supplementary Material, the authors refer to "yellow dashed lines". The dashed lines in Fig. 5 don't look yellow to me. This may just be a problem with my printer, but if they're not actually yellow, the authors should correct this.

The lines are yellow, but to avoid any confusion, we’ve changed it to simply “dashed lines”, since they are the only dashed lines in the figure.

11. In the first bit of text on page 6 of the Supplementary Material, the phrase "Z measurement followed by a Hadamard gate" should probably be reversed to become "Hadamard gate followed by a Z measurement" since it makes no sense to do a gate on a qubit that has been measured -- and because the effect of an X measurement (H followed by Z-meas.) is what is being described.

The referee is correct in stating that an X measurement is the same as a Hadamard followed by a Z measurement. However, in the particular case of X measurement of a qubit in a clique cluster state, an X measurement has the same effect as a Z measurement on a qubit and a Hadamard on one of its neighbors. In the previous version, it was unclear that the Z measurement and Hadamard were on different qubits. To prevent any confusion, we have rephrased the statement, and have also added a line pointing the reader to a reference which shows why this is true:

“The X measurement of a qubit in a clique removes the measured qubit and all its edges, and applies a Hadamard gate to one of the qubits that neighbored the measured qubit (before it was removed). This can be seen by looking at the set of stabilizers obtained when an a qubit in a clique is measured in the X basis as detailed in \cite{2015.PRA.Zaidi-Rudolph.BallisticLOQC}”

12. The authors should ensure that the abbreviations 'GCC' and 'LCC' have been properly defined before use in all sections of the main text and also separately in the Supplementary Material.

Please see the definitions in the response to question 8 above.

13. Typos
* (main) page 5, right column, near the top: "black bonds" should probably be "black nodes" (?)
* (main) page 6, left column, near middle: "it's lifetime" should be "its lifetime"
* (main) page 7, left column, near top: "high-Fidelity" should be "high-fidelity"
* (supplement) page 1, left column, near top: "if there are a path" should be "if there is a path"
* (supplement) page 2, left column, about 1/3 of the way down: within "L_{0k} = \{ v_{al}, v_{bl} \}", the subscript {bl} should probably be {bm} (I think).
* (supplement) page 2, left column, final paragraph: "if \{ v_a, v_b \} if" should be "if \{ v_a, v_b \} is"
* (supplement) page 2, right column, near top: The sentence "From Lemma 3..." is grammatically incorrect.
* (supplement) page 2, right column, about 2/3 of the way down: "every vertex is" should probably be "every vertex" (?)

We thank the referee for his careful reading and we have fixed all these typos.

14. Lemmas and Theorems in the Supplementary Material: The authors should use the 'amsthm' LaTeX package and its included {theorem}, {proof}, etc. environments. This ensures that lemmas and theorems are numbered uniquely throughout the document and that they and their proofs are formatted correctly (including ending proofs with a \qed symbol)

We are now using the {theorem} and {proof} environments in the proof.

Once these changes are properly addressed, I strongly recommend publication.

~~~~~~~~

Reviewer #3 (Remarks to the Author):

In the manuscript, titled “Percolation thresholds for photonic quantum computing”, authors studied linear optical quantum computing using the ballistic strategy and presented some interesting results.

Using linear optics to entangle photons is one of the most promising way of realising quantum computing. Preparing scalable cluster states is the critical step in linear optical quantum computing. Ballistic protocols are attractive protocols for preparing scalable cluster states. Ballistic protocols are not well studied as the alternative family of protocols, KLM and its variants. It is still an open question whether the performance of ballistic protocols can be superior to KLM family protocols. However, we know that KLM family protocols require a huge number of linear optical instruments [e.g. see PRX 5, 041007 (2015)]. It may turn out to be the case that the optimal protocol combines two strategies. General conclusions about the ballistic strategy are important.

The manuscript presented two general conclusions, which are the first two of four results as summarised by authors. However, these two conclusions are not sufficiently justified. Therefore, I do not recommend the publication of the manuscript.

About the first general conclusion:

The first general conclusion (main result 1) has two parts. The first part is a lower bound of the success probability threshold. If the success probability is higher than the threshold, scalable cluster states can be prepared using a ballistic protocol. The second part is a conjecture that the bound is tight. If the conjecture is true, a success probability higher than the lower bound is enough for preparing scalable cluster states.

All evidences supporting the second part are given in the appendix. I do not find that these evidences can support a reasonable conjecture accounted as a main result of a paper. I recommend authors to either move the second part from the first main result or provide more evidences. As a main result, authors should at least explain in the main text why they conjecture that the second part is true.

About the second general conclusion:

The second general conclusion may be more useful. Authors find that the success probability threshold of a ballistic
~~~~~~~~

protocol is the standard bound percolation threshold on a lattice. This result can help us in looking for the optimal protocol of linear optical quantum computing.

The second general conclusion is sound. However, why the bound percolation threshold of the fusion-operation lattice is the success probability threshold is not proved or explicitly explained. Actually, it may not be difficult to prove their conclusion. I recommend authors to explicitly give the proof rather than hand-waving.

We thank reviewer 3 for reading our manuscript carefully, and summarizing the main results. We have summarized our main results in the revised manuscript as follows:

1. We prove a general lower bound $\lambda_c^{(n)} > 1/(n-1)$, and provide evidence in favor of our conjecture that it is tight (achievable as closely as we wish).
2. We develop a systematic graphical construction of a universal cluster, wherein $\lambda_c^{(n)}$ becomes the usual bond percolation threshold of a logical graph that we specify, while accounting for the fusion failure modes. This is a key realization that was missing in both Kieling {\em et al.}'s construction~\cite{2007.PRL.Kieling-Eisert.PercolationQC} as well as in~\cite{2015.PRL.Gimeno-Segovia-Rudolph.3GHZtoBallisticQC, 2015.PRA.Zaidi-Rudolph.BallisticLOQC}. This not only enabled us to readily generate achievable thresholds (upper bounds on $\lambda_c^{(n)}$) using existing literature on bond percolation, but also helped us prove the aforesaid lower bound. We believe that this logical construction also naturally generalizes to future extensions that may employ multi-qubit fusions (projective measurements).
3. Using our logical construction, we prove $\lambda_c^{(3)} \le 0.5898$ as an achievable threshold, which improves upon the best existing result in~\cite{2015.PRL.Gimeno-Segovia-Rudolph.3GHZtoBallisticQC}. As a byproduct of our logical construction allowing a standard bond-percolation interpretation, we were able to reinterpret the construction of~\cite{2015.PRL.Gimeno-Segovia-Rudolph.3GHZtoBallisticQC} in our formalism and used Newman and Ziff's efficient Monte Carlo algorithm to refine their threshold~\eqref{eq:known_threshold2} to $\lambda_c^{(3)} \le 0.627$.
4. We settle a heretofore open question posed by Rudolph and collaborators~\cite{2015.PRL.Gimeno-Segovia-Rudolph.3GHZtoBallisticQC, 2015.PRA.Zaidi-Rudolph.BallisticLOQC, 2017.APLPhot.Rudolph.SiPhOptimism} by showing that a universal cluster can be created by linear-optically fusing 3-photon GHZ clusters over a particular 2D lattice with fusion success probability that is under what is theoretically achievable with an existing scheme~\cite{2014.PRL.Ewert-Loock.boostedfusion}, making this very attractive for integrated-photonic realizations.

In #1, the most important result is the general lower bound on the fusion success probability for the ballistic scheme to generate a resource renormalizable for universal quantum computing (meaning, the percolation condition be met for absolutely any way to fuse n-photon entangled clusters).

The conjecture that the bound is tight, is a conjecture, just like another conjecture in the paper that the regular bond percolation threshold p_c of any degree-n graph G is an achievable \lambda_c^{(n)}. This conjecture is not a main result of the paper, but we feel that the two pieces of evidence we present in its favor may provide intuition to the reader and motivate future work. We have also added a brief description of the evidence in the main section of the revised manuscript with further details in the supplementary information:

"This conjecture is supported by the fact that starting with N copies of photon clusters of size n and probabilistic operations that succeed with any probability greater than $1/(n-1)$, it is possible to obtain an $O(N)$ sized tree graph (detailed in supplementary information, section IIIA). We also present a random graph construction which provides intuition for why probabilistic operations on photon clusters of size n may provide enough connectivity to obtain a large connected cluster when $\lambda > 1/(n-1)$ (supplementary information, section IIIB)."

Since the time this paper was completed, we have significant progress on both of these conjectures – led by another student – which will hopefully be submitted as a standalone paper in the near future.

#2 is a major contribution of this paper. The realization that one can cast the ballistic cluster creation as bond percolation on an appropriate logical graph with certain properties, is an extremely important result. This not only let us reinterpret and refine the already-existing results from Rudolph, Keiling and co-authors, this let us discover better thresholds, which we doubt would have been found otherwise. This also let us employ a well known algorithm discovered by Newman and Ziff (for efficient evaluation of bond percolation thresholds) to find the fusion success

thresholds for ballistic cluster creation for photonic quantum computing. This let us refine previously published results, whose numerical simulations were extremely inefficient.

Our proof of achievability is rigorous. We substantially modified the section "C. Pure bond percolation on a logical graph" in the Supplemental Material, and would like to request reviewer 3 to read it and let us know if he/she finds the argument satisfactory.

#3 is our discovery of the best-known threshold for ballistic cluster creation using 3-photon GHZ clusters as a starting point. The individual two-photon fusion attempts needs to succeed with probability > 0.5898.

#4 is a very important contribution in our mind, particularly for experimental feasibility of this scheme – the proof that 3-photon GHZ clusters can be fused on a 2D logical lattice arrangement while meeting the percolation condition with a known linear-optical circuit design, is extremely important. Our 2D brickwork construction (Section II E of the main paper) needs fusion attempts to succeed with probability > 0.746, whereas there is a known circuit design (discovered a few years ago by Peter van Loock's group) which in principle can achieve a fusion success rate of 25/32 = 0.78125. Even though the margin between 0.78125 and 0.746 is not a lot (leaving little room for losses), this shows that a 2D architecture could in principle work. Whether this would be possible or not, was posed as a conjecture by Rudolph et al.

We believe that the above contributions are significant enough for this paper to be considered for publication. Clearly there is a lot to be done, and new results – by us or other groups – will merit new papers.

Other comments and questions:

1. It is costly to generate n-photon states starting from single photons. If this stage is considered, is it still that case that a larger n has advantage, because the lower bound decreases with n?
Generating n-photon states using emitters is also discussed in the manuscript. How good the emitters should be in terms of the loss rate and fidelity?

We have used a loss model that increases the loss per photon as n gets larger. We have added an explanation for this in section IIF. Quoting the revised text in the first paragraph of section IIF:

"We use a loss model inspired by proposals to produce photonic microclusters using quantum emitters~\cite{2009.PRL.Lindner-Rudolph.ClusMachGun, 2010.PRL.Economou-Rudolph.2DClusQD, 2015.PRB.Rao-Wrachtrup.DiamondEntPhotString} which have been recently realized experimentally~\cite{2016.Science.Schwartz-Gershoni.QDClusGen}. In these proposals, since each node of the microcluster is added sequentially, we assume that the total transmissivity of the photon in its lifetime is of the form $\eta = \eta_0^n$ (the loss is $1-\eta$) where n is the size of the microcluster and η_0 represents the transmissivity in one step. Hence, the total loss seen by the photon increases with the size of the microcluster. The import fidelity of the clusters generated due to unheralded photon losses and other forms of errors (e.g. mode mismatch, detector dark clicks) have not been considered."

2. "Seeing this requires a bit of imagination, and is not shown in Fig. 4." If it is not shown in the figure, authors should explain it a bit rather than rely on reader's imagination. On the diamond lattice, only red nodes are left, and each red node is connected to a neighbour red node with distance 3 in Fig. 4.

We have modified the following paragraph to be more explicit in describing why the post-fusion graph for the fusions done on the modified (10,3)-b lattice is a subgraph of the diamond lattice. Please see below.

In Fig.~\ref{fig:103b3D}, we use the same procedure to construct a 3D lattice with a lower percolation threshold. We place a 3-photon cluster at every node of this lattice and attempt fusions on its every bond. Since the black nodes have degree 3, all 3 photons of the clusters placed at those nodes get measured (and hence destroyed) in the process, whereas the red nodes, which have degree 2, are left with one unmeasured photon after all fusions have been attempted on their two neighboring bonds. Meeting the bond percolation condition of the modified $(10,3)$-b lattice ensures long-range connectivity. Yet, since the black nodes do not have any photons left over, the resulting photonic cluster state is a random sub graph of the effective lattice formed by the red nodes alone. This effective lattice happens to be the 3D diamond lattice. One can see this from the lattice in Fig.~\ref{fig:103b3D} as follows. Let us assume that all the fusion attempts succeed. The nodes of the left-over lattice will be the red nodes of the lattice in Fig.~\ref{fig:103b3D}. Let us pick any red node in Fig.~\ref{fig:103b3D} and follow the blue dashed edge up to the

neighboring black node. The black node has two two neighboring black nodes each of which is connected to a red node via blue dashed edges. Once the photons within the black nodes have disappeared after the fusions, the red node we started with will inherit these two red nodes as its immediate neighbors. Similarly, if we followed the blue dashed edge {\em down} from the original red node we picked, we would find two other red-node neighbors of that red node after the fusions are done. So, if all the fusion attempts were to succeed, we will get a regular 3D lattice of red nodes, where each node has degree 4. This is the diamond lattice. Therefore, if the fusions succeed with a probability $\lambda < 1$, the left over lattice will be a subgraph (percolated instance) of the diamond lattice. The authors of~\cite{2015.PRL.Gimeno-Segovia-Rudolph.3GHZtoBallisticQC} did exactly the above, attempting to create a percolated instance of the diamond lattice. But our re-interpretation of their (non-standard) percolation threshold as a standard bond percolation threshold of the modified $(10,3)$-b lattice not only provides a natural way to account for failure modes of fusion attempts, but allows one to exploit existing literature on efficient Monte Carlo methods (viz., the Newman-Ziff method~\cite{2001.PRE.Newman-Ziff.NZAlgo}) to calculate a sharper percolation threshold. More importantly, this interpretation also opens the door for new constructions and improved thresholds, which we describe next.

3. Usually, eta is used to denote efficiency rather than loss. Efficiency = 1 - Loss. Authors should clarify what eta means in the manuscript. For example, in the sentence "the total loss seen by a photon in it's lifetime is of the form eta = ...", eta is efficiency in the equation but it is called total loss. This question leads to another question. "a loss of 48.36% seen by each photon." Here, is 48.36% efficiency or loss? If it is efficiency and lower than 50%, does it mean that two scalable cluster states can be prepared rather than one using the protocol?

\eta is the transmissivity of the photon i.e. 1-loss. This has been clarified in the revised manuscript e.g. the statement quoted by the referee has been revised to "…we assume that the total transmissivity of the photon in its lifetime is of the form $\eta = \eta_0^n$ (the loss is $1-\eta$) where n is the size of the microcluster and η_0 represents the transmissivity in one step".

Regarding the referees second question, η_{0c} represents a bound on the loss, which has been clarified at the end of section IIF in the revised manuscript:

"Conversely, for a given fusion success probability λ, there exists a threshold η_{0c}, s.t., if $\eta_0 < \eta_{0c}$, the post-fusion cluster cannot be percolated. We thus have a lower bound $\eta_{0c} \ge \eta_{0c}^{\rm{(LB)}}$, where $\eta_{0c}^{\rm{(LB)}} = \left[1/{\lambda(n-1)}\right]^{1/(2n)}$. In Fig.~\ref{fig:phloss}(b), we plot $\eta_{0c}^{\rm (LB)}$ for different values of n, for $\lambda = 0.75$ and $\lambda = 1$. There is an optimum value of n which gives the best lower bound on loss tolerance, e.g., for $\lambda = 0.75$, six photon microclusters gives the lowest bound of $\eta_{0c}^{\rm{(LB)}} = 0.8957$ which corresponds to a loss (1-η) of 48.36% seen by each photon. Furthermore, we find that going from $\lambda = 0.75$, which is attainable using four single ancilla photons and (lossless) linear optics~\cite{2014.PRL.Ewert-Loock.boostedfusion} to deterministic fusion ($\lambda = 1$), $\eta_{0c}^{\rm{(LB)}}$ only decreases slightly, i.e., the equivalent per-photon loss threshold increases from $~89.6 \%$ to $~87.1 \%$. Hence, when losses are accounted for in ballistic cluster state creation, the advantage in having a fully deterministic fusion may be relatively small. It's important to note, however, the numbers presented here are only lower bounds on η_{0c} and λ_{0c}, and may not be tight. For example, our results show that any ballistic cluster creation process that starts with six photon microclusters and uses destructive fusion with $\lambda = 0.75$ cannot tolerate more than 48.36% loss. However, these results do not prove that 48.36% loss is sufficient for ballistic cluster creation."

REVIEWERS' COMMENTS:

Reviewer #2 (Remarks to the Author):

The authors have addressed all of my criticisms and concerns, and they appear to have done the same with respect to those of the other referee.

I recommend publication.

Reviewer #3 (Remarks to the Author):

In the previous report, my main concern is that one of the main results, i.e., result #2, is not rigorously proved. I do not think the conclusion is incorrect. Therefore, I am not opposed to the publication of the paper. However, I do think a proof of result #2 is still missing in the revised manuscript.

To be specific, for enough justification, the following components are necessary:
1. A general map from a logical graph to the graph of a graph state, for any logical graph;
2. This map explicitly corresponds to a fusion protocol, i.e., the graph state is a result of fusions according to the logical graph;
3. The percolation of the logical graph is mapped to the percolation of the graph state, for any logical graph.

These components may already exist in the current version. However, I cannot find. If this is the case, readers could benefit from a more compact organisation of the proof.

All other questions have been sufficiently addressed in the response and revised manuscript.

Reviewer #3 (Remarks to the Author):

In the previous report, my main concern is that one of the main results, i.e., result #2, is not rigorously proved. I do not think the conclusion is incorrect. Therefore, I am not opposed to the publication of the paper. However, I do think a proof of result #2 is still missing in the revised manuscript.

To be specific, for enough justification, the following components are necessary:

1. A general map from a logical graph to the graph of a graph state, for any logical graph; 2. This map explicitly corresponds to a fusion protocol, i.e., the graph state is a result of fusions according to the logical graph; 3. The percolation of the logical graph is mapped to the percolation of the graph state, for any logical graph.

These components may already exist in the current version. However, I cannot find. If this is the case, readers could benefit from a more compact organisation of the proof.

All other questions have been sufficiently addressed in the response and revised manuscript.

We took a careful read of the logical graph to photonic graph mapping, and we do realize why reviewer 3 has been dissatisfied with the level of rigor. We apologize, and thank the reviewer for pointing this out, and stressing on improving the description of the mapping. We completely re-wrote Section II C "Reconciling prior results in a new framework", aided by our revised Fig. 4, which we hope will be much clearer than before. Our mapping—as described in the re-written Section IIC—is rigorous and sufficient for all the examples we consider in the paper, i.e., involving three-photon clusters. However, per reviewer 3's suggestion, we have added the following sentence at the end of the second point under Main results summary:

"However, developing a fully axiomatic construction of this logical graph for the most general case if left open for future work."

We believe the changes address the final concerns raised by the reviewer. We thank the reviewer again, for the comments we believe has made the manuscript better.
