# Supplementary material for: Percolation thresholds for photonic quantum computing
Source: Nat Commun. 2019 Mar 6;10:1070. doi: 10.1038/s41467-019-08948-x (PMC6403388; doi:10.1038/s41467-019-08948-x)
Supplement: Supplementary file 2 — Peer Review File [file 41467_2019_8948_MOESM2_ESM.pdf]

Supplementary Information: Percolation thresholds for photonic quantum computing

Pant et al.

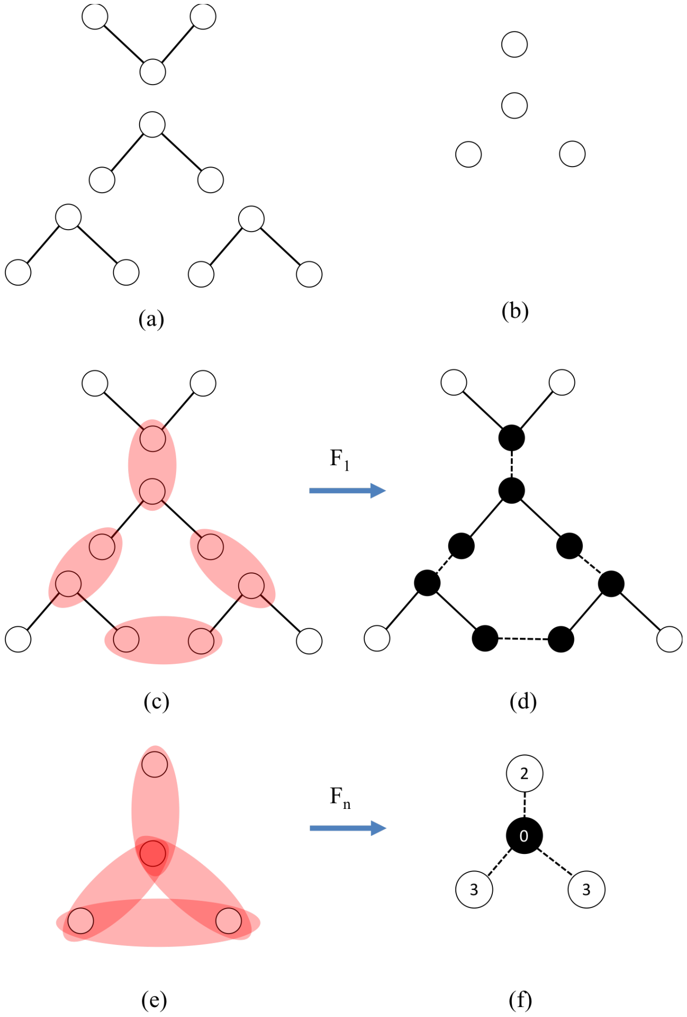

Supplementary Figure 1. (a) Graph G_0 and (b) Graph G_1 correspoing to G_0. (c) Pairs of vertices of graph G_0 inside the red ovals are contained in list $\vec{\mathbf{L}}_0$. (d) The resulting graph when F_1 has inputs G_0 and $\vec{\mathbf{L}}_0$ as specified in (c). The dashed lines are present if and only if the corresponding entry in $\vec{\mathbf{S}}$ is 1. (e) Pairs of vertices of graph G_1 inside the red ovals are contained in list $\vec{\mathbf{L}}_1$ derived from $\vec{\mathbf{L}}_0$. (f) The resulting graph when F_n has inputs G_1 and $\vec{\mathbf{L}}_1$ as specified in (e). The dashed lines are present if and only if the corresponding entry in $\vec{\mathbf{S}}$ is 1.

SUPPLEMENTARY NOTE 1. CONVERSE PROOFS

A. Proof of $\lambda_c^{(n)} \geq 1/(n-1)$

Theorem 1. *Starting with N isolated clusters of n vertices each where n is finite, the size of the largest connected component obtained from any set of attempted "destructive two-node fusions" that succeed with probability λ is $o(N)$ for $\lambda < 1/(n-1)$.*

Proof outline: A 'destructive two-node" fusion (D in the main proof) is a function whose input is a graph G_0, a list of pairs of vertices $\vec{\mathbf{L}}_0$ which represents the vertices undergoing fusion, and a binary list $\vec{\mathbf{S}}$ which represents which of the fusions is "successful" (and hence has the same number of entries as $\vec{\mathbf{L}}_0$). The output is another graph in which the nodes contained in $\vec{\mathbf{L}}_0$ are removed, and some edges may be added depending on $\vec{\mathbf{S}}$ (explained in the main proof). The linear optic fusions studied in this paper are destructive two-nodes fusions. Supplementary Fig. 1(a) shows an example of input graph G_0 with $n = 3$ sized states and the red ovals in Supplementary Fig. 1(c) contain pairs of vertices that are the elements of $\vec{\mathbf{L}}_0$. We also define a new graph operation F_1 that has the same inputs as D. The output graph of F_1 has the same vertices as the input graph G_0 but the vertices contained in $\vec{\mathbf{L}}_0$ are colored black

as shown in Supplementary Fig. 1(d). Furthermore, we place an edge between the vertices contained in an entry in $\vec{\mathbf{L}}_0$ if the corresponding entry in $\vec{\mathbf{S}}$. If F_1 acts on the inputs depicted in Supplementary Fig. 1(c), the output graph is Supplementary Fig. 1(d) with a dashed edge present if the corresponding entry in $\vec{\mathbf{S}}$ is 1, and absent otherwise. Each node can be input to only a single operation in F_1 (in other words, only uncolored nodes can undergo an F_1 operation). We first show that the largest connected component (LCC) obtained after a series of operations F_1 on G_0 is larger than or equal to the LCC obtained after the corresponding series of destructive two-node fusions D (Lemma 1).

We then map the graph G_0 (Supplementary Fig. 1(a)) to a graph G_1 (Supplementary Fig. 1(b)). Each isolated size cluster of n vertices in G_0 is replaced by a single node in G_1. Furthermore, we map the list $\vec{\mathbf{L}}_0$ to a new list $\vec{\mathbf{L}}_1$ in which every node in G_0 is replaced by the corresponding node in G_1. Supplementary Fig. 1(e) shows red ovals corresponding to $\vec{\mathbf{L}}_1$ acting on G_0. We define a function F_n that is the same as F_1, except that each node can now undergo n operations. Supplementary Fig. 1(f) shows the output graph when F_n has G_1, $\vec{\mathbf{L}}_1$ as inputs. The dashed lines are present if and only if the corresponding entry in $\vec{\mathbf{S}}$ is 1. The numbers inside the vertices track the number of times a node has undergone fusion and if the number reaches $n = 3$, we color it black (no more fusions involving a black node can be attampted). We show that the size of the LCC obtained after F_n acting on G_0 is within a constant factor of the the LCC obtained when F_1 acts on G_0 (Lemma 2).

Finally, since F_n acting on G_1 is an instance of regular bond percolation, we bound the minimum success probability required for the graph operation to obtain an LCC proportional to the size of G_0 using results from classical percolation theory (Lemma 3).

Formal proof: We now present the proof and formally define the operations D, F_n:

A graph $\mathrm{G} = (\mathrm{V}, \mathrm{E})$ is a collection of vertices V and edges E with each vertex having a color property that is a whole number. Two vertices in a graph are said to be connected if there is a path connecting them. LCC(G) is the number of vertices in the largest connected component in G.

We define a function $\mathrm{G}' = \mathrm{F}_n(\mathrm{G}, L, S)$ with $n \geq 1$ that has the following inputs: G is the input graph, $\mathrm{L} = \{\mathrm{u}, \mathrm{w}\}$ is a set of two vertices in G and $S \in \{0, 1\}$. L is a valid input only if both u and w have nonzero color. G' is a graph that is identical to G except for the following differences: 1) The colors of u and w are incremented by one modulo $n + 1$, 2) If $S = 1$ and $\mathrm{u} \neq \mathrm{w}$, then an edge is introduced between u and w. We use $\mathrm{F}_n(\mathrm{G}, \vec{\mathbf{L}}, \vec{\mathbf{S}})$ with input lists $\vec{\mathbf{L}} = \{\mathrm{L}_1, \mathrm{L}_2, \ldots, \mathrm{L}_k\}$ and $\vec{\mathbf{S}} = \{S_1, S_2, \ldots, S_k\}$ as shorthand for $\mathrm{F}_n(\ldots \mathrm{F}_n(\mathrm{F}_n(\mathrm{G}, \mathrm{L}_1, S_1), \mathrm{L}_2, S_2), \ldots), \mathrm{L}_k, S_k)$. Based on this definition of $\mathrm{F}_n(\mathrm{G}, \vec{\mathbf{L}}, \vec{\mathbf{S}})$, if all vertices in G have color 1, $\vec{\mathbf{L}}$ is a valid list if and only if every vertex appears no more than n times in $\vec{\mathbf{L}}$

We now define a class of functions that we call "destructive two node fusions". A "destructive two node fusion" is a function of the form $\mathrm{G}' = \mathrm{D}(\mathrm{G}, \mathrm{L}, S)$ where G is the input graph, $\mathrm{L} = \{\mathrm{u}, \mathrm{w}\}$ is a set of two vertices in G and $S \in \{0, 1\}$. G' is the output graph which has the same set of vertices as G but different vertex colorings and a different set of edges. L is a valid input only if both u and w have nonzero color in G. U and W are the set of vertices in G connected to u and w respectively. The function $\mathrm{G}' = \mathrm{D}(\mathrm{G}, \mathrm{L}, S)$ is a "destructive two node fusion" if and only if it follows the following rules: *Rule 1* In G', u and w have color 0 and no edges attached to them. *Rule 2* If vertex $\mathrm{a} \notin \mathrm{U} \cup \mathrm{W}$ was not connected to a vertex b in G, a and b should remain unconnected in G'. *Rule 3* For $S = 0$ and $\mathrm{U} \neq \mathrm{W}$, every vertex in U is disconnected from every vertex in W. As in the case for F_n, $\mathrm{D}(\mathrm{G}, \vec{\mathbf{L}}, \vec{\mathbf{S}})$ with $\vec{\mathbf{L}} = \{\mathrm{L}_1, \mathrm{L}_2, \ldots, \mathrm{L}_k\}$ and $\vec{\mathbf{S}} = \{S_1, S_2, \ldots, S_k\}$ is shorthand for $\mathrm{D}(\ldots \mathrm{D}(\mathrm{D}(\mathrm{G}, \mathrm{L}_1, S_1), \mathrm{L}_2, S_2), \ldots), \mathrm{L}_k, S_k)$. It should be noted that a set of valid inputs G, $\vec{\mathbf{L}}$, $\vec{\mathbf{S}}$ to $\mathrm{D}(\mathrm{G}, \vec{\mathbf{L}}, \vec{\mathbf{S}})$ is always a valid input to $\mathrm{F}_1(\mathrm{G}, \tilde{\mathbf{L}}, \tilde{\mathbf{S}})$ as well. Also, note that F_n is not in the class of "destructive two node fusion".

Lemma 1. *$LCC(F_1(G, \vec{\mathbf{L}}, \vec{\mathbf{S}})) \geq LCC(D(G, \vec{\mathbf{L}}, \vec{\mathbf{S}}))$ for any "destructive two node fusion" D and any set of valid inputs to D.*

Proof. Consider 2 graphs $\mathrm{G_i}$ and $\mathrm{G_j}$ with the same vertices and vertex colorings. Connectivity between any two vertices in $\mathrm{G_i}$ implies connectivity between the same vertices in $\mathrm{G_j}$. u and w are vertices in $\mathrm{G_i}$, $\mathrm{G_j}$ and U and W are the set of vertices in $\mathrm{G_i}$ connected to u and w respectively. D is a "destructive two node fusion". If u and w are connected in $\mathrm{G_D} = \mathrm{D}(\mathrm{G}_i, \{\mathrm{a}, \mathrm{b}\}, S)$, either u and w are connected in $\mathrm{G_i}$ or $(S = 1)\&((\mathrm{a} \in \mathrm{U}\&\mathrm{b} \in \mathrm{W}) \vee (\mathrm{a} \in \mathrm{W}\&\mathrm{b} \in \mathrm{U}))$. If u and w are connected in $\mathrm{G_i}$, they must be connected in $\mathrm{G_F} = \mathrm{F}_1(\mathrm{G_j}, \{\mathrm{a}, \mathrm{b}\}, S)$ since connectivity in $\mathrm{G_i}$ implies connectivity in $\mathrm{G_j}$ and F_1 does not remove edges. If $(S = 1)\&((\mathrm{a} \in \mathrm{U}\&\mathrm{b} \in \mathrm{W}) \vee (\mathrm{a} \in \mathrm{W}\&\mathrm{b} \in \mathrm{U}))$, there is an edge between a vertex of U and a vertex of W in $\mathrm{G_F}$ which implies that u and w are connected. Therefore, connectivity of u and w in $\mathrm{G_D}$ implies connectivity of u and w in $\mathrm{G_F}$.

Consider a graph G with two vertices u, w. Using the result of the previous paragraph with $\mathrm{G_i} = \mathrm{G_j} = \mathrm{G}$, connectivity of u, w in $\mathrm{G_{D1}} = \mathrm{D}(\mathrm{G}, \mathrm{L}_0, S_0)$ implies connectivity of u, w in $\mathrm{G_{F1}} = \mathrm{F}_1(\mathrm{G}, \mathrm{L}_0, S_0)$. Using the result from the previous paragraph again with $\mathrm{G_i} = \mathrm{G_{D1}}$ and $\mathrm{G_j} = \mathrm{G_{F1}}$, connectivity of u, w in $\mathrm{G_{D2}} = D(\mathrm{G_{D1}}, \mathrm{L}_1, S_1)$ implies connectivity of u, w in $\mathrm{G_{F2}} = \mathrm{F}_1(\mathrm{G_{F1}}, \mathrm{L}_1, S_1)$. By repeating this process with lists $\vec{\mathbf{L}} = (\mathrm{L}_1, \mathrm{L}_2, \ldots, \mathrm{L}_k)$ and

$\vec{\mathbf{S}} = (S_1, S_2, \ldots, S_k)$, if two vertices are connected in $\mathrm{D}(\mathrm{G}, \vec{\mathbf{L}}, \vec{\mathbf{S}})$, they are connected in $\mathrm{F}_1(\mathrm{G}, \vec{\mathbf{L}}, \vec{\mathbf{S}})$. This leads to Lemma 1. □

G_0 is a graph composed of nN vertices which are labelled as v_{lr} with $l \in 1, 2, \ldots, N$ and $r \in \{1, 2, \ldots, n\}$. Two vertices v_{lr} and $\mathrm{v}_{l'r'}$ are unconnected if $l \neq l'$. We define C_k as the set of all vertices of the form v_{kr} with $r \in \{1, 2, \ldots n\}$. Hence, G_0 is composed of N isolated clusters labelled as $\mathrm{C}_1, \mathrm{C}_2, \ldots, \mathrm{C}_N$. All vertices of G_0 have color 1. Note that two vertices in the same cluster may or may not be connected but vertices in different clusters are unconnected.

G_1 is a graph that consists of N vertices labelled as v_l with $l \in \{1, 2, \ldots, N\}$. Every vertex v_k of G_1 maps to cluster C_k in G_0. There are no edges in G_1. All vertices of G_1 have color 1.

$\vec{\mathbf{L}}_0$ is a list that is valid as input to $\mathrm{F}_1(\mathrm{G}_0, \vec{\mathbf{L}}_0, \vec{\mathbf{S}})$. This requires that every vertex appears no more than once in $\vec{\mathbf{L}}_0$. $\vec{\mathbf{L}}_1(\vec{\mathbf{L}}_0)$ maps the list $\vec{\mathbf{L}}_0$, which contains vertices from G_0 to a list that contains vertices in G_1 by removing the second subscript in every vertex e.g. if $\vec{\mathbf{L}}_0 = (\{\mathrm{v}_{11}, \mathrm{v}_{22}\}, \{\mathrm{v}_{12}, \mathrm{v}_{31}\}, \ldots)$, $\vec{\mathbf{L}}_1(\vec{\mathbf{L}}_0) = (\{\mathrm{v}_1, \mathrm{v}_2\}, \{\mathrm{v}_1, \mathrm{v}_3\}, \ldots)$.

Lemma 2. *$n LCC(F_n(G_1, \vec{\mathbf{L}}_1(\vec{\mathbf{L}}_0), \vec{\mathbf{S}})) \geq LCC(F_1(G_0, \vec{\mathbf{L}}_0, \vec{\mathbf{S}}))$ for any valid list $\vec{\mathbf{L}}_0$.*

Proof. For $\vec{\mathbf{L}}_0$ to be a valid input to $\mathrm{F}_1(\mathrm{G}_0, \vec{\mathbf{L}}_0, \vec{\mathbf{S}})$, every vertex appears at most once in it. n vertices from G_0 in $\vec{\mathbf{L}}_0$ map to 1 vertex from G_1 in $\vec{\mathbf{L}}_1(\vec{\mathbf{L}}_0)$. Hence, every vertex appears at most n times in $\vec{\mathbf{L}}_1(\vec{\mathbf{L}}_0)$ and therefore $\vec{\mathbf{L}}_1(\vec{\mathbf{L}}_0)$ is a valid input to $\mathrm{F}_n(\mathrm{G}_1, \vec{\mathbf{L}}_1(\vec{\mathbf{L}}_0), \vec{\mathbf{S}})$.

If two vertices from different clusters C_a and C_b in $\mathrm{F}_1(\mathrm{G}_0, \vec{\mathbf{L}}_0, \vec{\mathbf{S}})$ are connected, there must exist at least one element $\mathrm{L}_{0k} = \{\mathrm{v}_{al}, \mathrm{v}_{br}\}$ in $\vec{\mathbf{L}}_0$ with the corresponding entry in $\vec{\mathbf{S}}$, $S_k = 1$. $l, r \in 1, 2, \ldots, n$. Hence the corresponding entry in $\vec{\mathbf{L}}_1(\vec{\mathbf{L}}_0)$ must have the form $\mathrm{L}_{1k} = \{\mathrm{v}_a, \mathrm{v}_b\}$ with $S_k = 1$. Therefore, if two vertices in two different clusters C_a and C_b are connected in $\mathrm{F}_1(\mathrm{G}_0, \vec{\mathbf{L}}_0, \vec{\mathbf{S}})$, the corresponding vertices v_a and v_b in $\mathrm{F}_n(\mathrm{G}_1, \vec{\mathbf{L}}_1(\vec{\mathbf{L}}_0), \vec{\mathbf{S}})$ are connected.

Suppose that the largest connected component of $\mathrm{F}_1(\mathrm{G}_0, \vec{\mathbf{L}}_0, \vec{\mathbf{S}})$ contains vertices from k different clusters. Since the size of every cluster is n, this implies that $\mathrm{LCC}(\mathrm{F}_1(\mathrm{G}_0, \vec{\mathbf{L}}_0, \vec{\mathbf{S}})) \leq nk$ with the equality holding if any two vertices within the same cluster are connected. From the previous paragraph, if any vertex in $\mathrm{F}_1(\mathrm{G}_0, \vec{\mathbf{L}}_0, \vec{\mathbf{S}})$ is a part of the largest connected component, the corresponding vertex in $\mathrm{F}_n(\mathrm{G}_1, \vec{\mathbf{L}}_1(\vec{\mathbf{L}}_0), \vec{\mathbf{S}})$ will be part of the largest connected component. Hence, $\mathrm{LCC}(\mathrm{F}_n(\mathrm{G}_1, \vec{\mathbf{L}}_1(\vec{\mathbf{L}}_0))) = k$. Therefore, $n\mathrm{LCC}(\mathrm{F}_n(\mathrm{G}_1, \vec{\mathbf{L}}_1(\vec{\mathbf{L}}_0))) = nk \geq \mathrm{LCC}(\mathrm{F}_1(\mathrm{G}_0, \vec{\mathbf{L}}_0, \vec{\mathbf{S}}))$ and we obtain Lemma 2. □

Assume that $\vec{\mathbf{L}}_2$, a list of node pairs from G_1 is a valid input to $\mathrm{F}_n(\mathrm{G}_1, \vec{\mathbf{L}}_2, \vec{\mathbf{S}})$.

Lemma 3. *For $\lambda < 1/(n-1)$, $LCC(F_n(G_1, \vec{\mathbf{L}}_2, \vec{\mathbf{S}}(\lambda))) = o(N)$ for any valid list $\vec{\mathbf{L}}_2$ where $\vec{\mathbf{S}}(\lambda)$ is a probabilistic function that returns a string of bits of the same size as the number of elements in $\vec{\mathbf{L}}_2$. Each entry of $\vec{\mathbf{S}}(\lambda)$ is independently chosen as 1 with probability λ and 0 with probability $1 - \lambda$.*

Proof. The graph $\mathrm{F}_n(\mathrm{G}_1, \vec{\mathbf{L}}_2, \vec{\mathbf{S}}(\lambda))$ contains an edge between two vertices v_a and v_b if and only if $\{v_a, v_b\}$ is an element of $\vec{\mathbf{L}}_2$ and the corresponding bit in $\vec{\mathbf{S}}(\lambda)$ is 1. Since the entries of $\vec{\mathbf{S}}(\lambda)$ are chosen independently as 1 with probability λ, $\mathrm{F}_n(\mathrm{G}_1, \vec{\mathbf{L}}_2, \vec{\mathbf{S}}(\lambda))$ represents an instance of bond percolation with bond probability λ over the set of vertices in G_1 with the set of attempted edges defined by the elements of $\vec{\mathbf{L}}_2$. For $\vec{\mathbf{L}}_2$ to be a valid list, a vertex can appear in $\vec{\mathbf{L}}_2$ no more than n times which implies that the attempted graph of this bond percolation instance has maximum degree n. For bond percolation on a graph with maximum degree n, if the bond probability is less than $1/(n-1)$, with high probability, the largest connected component is of size $o(N)$ [1, 2]. This proves Lemma 3. □

From Lemma 1, $\mathrm{LCC}(\mathrm{D}(\mathrm{G}_0, \vec{\mathbf{L}}_0, \vec{\mathbf{S}}(\lambda))) \leq \mathrm{LCC}(\mathrm{F}_1(\mathrm{G}_0, \vec{\mathbf{L}}_0, \vec{\mathbf{S}}(\lambda)))$ for any "destructive two node fusion" D. This implies, from Lemma 2 that $\mathrm{LCC}(\mathrm{D}(\mathrm{G}_0, \vec{\mathbf{L}}_0, \vec{\mathbf{S}}(\lambda))) \leq \mathrm{LCC}(\mathrm{F}_1(\mathrm{G}_0, \vec{\mathbf{L}}_0, \vec{\mathbf{S}}(\lambda))) \leq n\mathrm{LCC}(\mathrm{F}_n(\mathrm{G}_1, \vec{\mathbf{L}}_1(\vec{\mathbf{L}}_0), \vec{\mathbf{S}}(\lambda)))$ where G_0, G_1, $\vec{\mathbf{L}}_0$ and $\vec{\mathbf{L}}_1(\vec{\mathbf{L}}_0)$ are described in the lead-up to Lemma 2. Using Lemma 3, $\mathrm{LCC}(\mathrm{F}_n(\mathrm{G}_1, \vec{\mathbf{L}}_1(\vec{\mathbf{L}}_0), \vec{\mathbf{S}}(\lambda))) = o(N)$ if $\lambda < 1/(n-1)$, and we obtain Theorem 1 stated at the beginning of this note. In other words, $\lambda_{\mathrm{c}}^{(n)} \geq 1/(n-1)$

B. Generalization to m node fusions: Proof of $\lambda_{\mathrm{c}}^{(n,m)} \geq 1/[(n-1)(m-1)]$

Theorem 2. *Starting with N isolated clusters of n vertices each where n is finite, the size of the largest connected component obtained from any set of attempted "destructive m-node fusions" that succeed with probability λ is $o(N)$ for $\lambda < 1/[(n-1)(m-1)]$.*

This subsection closely resembles the proof of the lower bound for two node fusions.

We generalize the bound to the following definition of m node fusions: A "destructive m node fusion" is a function of the form $\mathrm{G}' = \mathrm{D}^{(m)}(\mathrm{G}, \mathrm{L}, S)$ where G is the input graph, $\mathrm{L} = \{\mathrm{u}_1, \mathrm{u}_2, \ldots, \mathrm{u}_m\}$ is a set of m vertices in G and $S \in \{0, 1\}$. G' is the output graph which has the same set of vertices as G but different vertex colorings and a different set of edges. L is a valid input if and only if $\mathrm{u}_1, \mathrm{u}_2, \ldots, \mathrm{u}_m$ are all different and all have nonzero color. $\mathrm{U}_1, \mathrm{U}_2, \ldots, \mathrm{U}_m$ are the set of vertices in G connected to $\mathrm{u}_1, \mathrm{u}_2, \ldots, \mathrm{u}_m$ respectively. The function $\mathrm{G}' = \mathrm{D}^{(m)}(\mathrm{G}, \mathrm{L}, S)$ is a "destructive m node fusion" if and only if it follows the following rules: *Rule 1* In G', $\mathrm{u}_1, \mathrm{u}_2, \ldots, \mathrm{u}_m$ all have color 0 and no edges attached to them. *Rule 2* If vertex $\mathrm{a} \notin \bigcup_{k=1}^{m} \mathrm{U}_k$ was not connected to a vertex b in G, a and b should remain unconnected in G'. *Rule 3* For $S = 0$ and $\mathrm{U}_i \neq \mathrm{U}_j$, every vertex in U_i is disconnected from every vertex in U_j in G' $\forall i, j \in \{1, 2, \ldots, m\}$. As in the previous proof, $\mathrm{D}^{(m)}(\mathrm{G}, \vec{\mathbf{L}}, \vec{\mathbf{S}})$ with $\vec{\mathbf{L}} = \{\mathrm{L}_1, \mathrm{L}_2, \ldots, \mathrm{L}_k\}$ and $\vec{\mathbf{S}} = \{S_1, S_2, \ldots, S_k\}$ is shorthand for $\mathrm{D}^{(m)}(\ldots \mathrm{D}^{(m)}(\mathrm{D}^{(m)}(\mathrm{G}, \mathrm{L}_1, S_1), \mathrm{L}_2, S_2), \ldots), \mathrm{L}_k, S_k)$. In our definition of an m node fusion, a failed fusion attempt never connects nodes that were unconnected in the input graph.

We also define function $\mathrm{G}' = \mathrm{F}_n^{(m)}(\mathrm{G}, \mathrm{L}, S)$ analogous to $\mathrm{F}_n(\mathrm{G}, \mathrm{L}, S)$ with $n \geq 1$ that has the following inputs: G is the input graph, $\mathrm{L} = \{\mathrm{u}_1, \mathrm{u}_2, \ldots, \mathrm{u}_m\}$ is a set of m vertices in G and $S \in \{0, 1\}$. L is a valid input if and only if $\mathrm{u}_1, \mathrm{u}_2, \ldots, \mathrm{u}_m$ are all different and all have nonzero color. G' is a graph that is identical to G except for the following differences: 1) The colors of $\mathrm{u}_1, \mathrm{u}_2, \ldots, \mathrm{u}_m$ are all incremented by one modulo $n + 1$, 2) If $S = 1$, a vertex w with color 0 is added to the graph and an edge is introduced between w and $\mathrm{u}_k \forall k \in \{1, 2, \ldots, d\}$ (let's call them connection nodes). We use $\mathrm{F}_n^{(m)}(\mathrm{G}, \vec{\mathbf{L}}, \vec{\mathbf{S}})$ with input lists $\vec{\mathbf{L}} = \{\mathrm{L}_1, \mathrm{L}_2, \ldots, \mathrm{L}_k\}$ and $\vec{\mathbf{S}} = \{S_1, S_2, \ldots, S_k\}$ as shorthand for $\mathrm{F}_n^{(m)}(\ldots \mathrm{F}_n^{(m)}(\mathrm{F}_n^{(m)}(\mathrm{G}, \mathrm{L}_1, S_1), \mathrm{L}_2, S_2), \ldots), \mathrm{L}_k, S_k)$. Based on this definition of $\mathrm{F}_n^{(m)}(\mathrm{G}, \vec{\mathbf{L}}, \vec{\mathbf{S}})$, if all vertices in G have color 1, $\vec{\mathbf{L}}$ is a valid list if and only if every vertex appears no more than n times in $\vec{\mathbf{L}}$

A set of valid inputs G, $\vec{\mathbf{L}}$, $\vec{\mathbf{S}}$ to $\mathrm{D}^{(m)}(\mathrm{G}, \vec{\mathbf{L}}, \vec{\mathbf{S}})$ is always a valid input to $\mathrm{F}_1^{(m)}(\mathrm{G}, \vec{\mathbf{L}}, \vec{\mathbf{S}})$ as well. Note that $\mathrm{F}_n^{(m)}$ is not a "destructive m node fusion".

Lemma 4. *$LCC(F_1^{(m)}(G, \vec{\mathbf{L}}, \vec{\mathbf{S}})) \geq LCC(D^{(m)}(G, \vec{\mathbf{L}}, \vec{\mathbf{S}}))$ for any "destructive m node fusion" D and any set of valid inputs to D.*

Proof. Consider 2 graphs $\mathrm{G_i}$ and $\mathrm{G_j}$ with the same vertices and vertex colorings. Connectivity between any two vertices in $\mathrm{G_i}$ implies connectivity between the same vertices in $\mathrm{G_j}$. a, b are vertices in $\mathrm{G_i}$, $\mathrm{G_j}$ and A, B are the set of vertices in $\mathrm{G_i}$ connected to a and b respectively. $\mathrm{D}^{(m)}$ is a "destructive m node fusion". If a and b are connected in $\mathrm{G_D} = \mathrm{D}(\mathrm{G}_i, \mathrm{L}, S)$ with $\mathrm{L} = \{\mathrm{u}_1, \mathrm{u}_2, \ldots, \mathrm{u}_m\}$, either a and b are connected in $\mathrm{G_i}$ or $(S = 1)\&(\mathrm{u}_k \in \mathrm{A}\&\mathrm{u}_l \in \mathrm{B})$ for some $k, l \in 1, 2, \ldots, m$. If a and b are connected in $\mathrm{G_i}$, they must be connected in $\mathrm{G_F} = \mathrm{F}_1^{(m)}(\mathrm{G}_j, \mathrm{L}, S)$ since connectivity in $\mathrm{G_i}$ implies connectivity in $\mathrm{G_j}$ and $\mathrm{F}_1^{(m)}$ does not remove edges. If $(S = 1)\&(\mathrm{u}_k \in \mathrm{A}\&\mathrm{u}_l \in \mathrm{B})$, a vertex of A and a vertex of B share a vertex w as a neighbor in $\mathrm{G_F}$ which implies that a and b are connected. Therefore, connectivity of a and b in $\mathrm{G_D}$ implies connectivity of a and b in $\mathrm{G_F}$.

Consider a graph G with two vertices a, b. Using the result of the previous paragraph with $\mathrm{G_i} = \mathrm{G_j} = \mathrm{G}$, connectivity of a, b in $\mathrm{G_{D1}} = \mathrm{D}^{(m)}(\mathrm{G}, \mathrm{L}_1, S_1)$ implies connectivity of a, b in $\mathrm{G_{F1}} = \mathrm{F}_1^{(m)}(\mathrm{G}, \mathrm{L}_1, S_1)$. Using the result from the previous paragraph again with $\mathrm{G}_i = \mathrm{G_{D1}}$ and $\mathrm{G_j} = \mathrm{G_{F1}}$, connectivity of a, c in $\mathrm{G_{D2}} = \mathrm{D}^{(m)}(\mathrm{G_{D1}}, \mathrm{L}_2, S_2)$ implies connectivity of a, b in $\mathrm{G_{F2}} = \mathrm{F}_n^{(m)}(\mathrm{G_{F1}}, \mathrm{L}_2, S_2)$. By repeating this process with lists $\vec{\mathbf{L}} = (\mathrm{L}_1, \mathrm{L}_2, \ldots, \mathrm{L}_k)$ and $\vec{\mathbf{S}} = (S_1, S_2, \ldots, S_k)$, if two vertices are connected in $\mathrm{D}^{(m)}(\mathrm{G}, \vec{\mathbf{L}}, \vec{\mathbf{S}})$, they are connected in $\mathrm{F}_1^{(m)}(\mathrm{G}, \vec{\mathbf{L}}, \vec{\mathbf{S}})$. This leads to Lemma 4. □

G_0 is a graph composed of nN vertices which are labelled as v_{lr} with $l \in 1, 2, \ldots, N$ and $r \in \{1, 2, \ldots, n\}$. Two vertices v_{lr} and $\mathrm{v}_{l'r'}$ are unconnected if $l \neq l'$. We define cluster C_k as the set of all vertices of the form v_{kr} with $r \in \{1, 2, \ldots n\}$. Hence, G_0 is composed of N isolated clusters $\mathrm{C}_1, \mathrm{C}_2, \ldots, \mathrm{C}_N$. All vertices have color 1. Note that two vertices in the same cluster may or may not be connected but vertices in different clusters are unconnected.

$\mathrm{G}_1(\mathrm{G}_0)$ is a function that takes graph G_0 as input and returns a graph with N vertices labelled as v_l with $l \in \{1, 2, \ldots, N\}$. Every vertex v_k of $\mathrm{G}_1(\mathrm{G}_0)$ maps to cluster C_k in G_0. There are no edges in $\mathrm{G}_1(\mathrm{G}_0)$ and all vertices have color 1.

$\vec{\mathbf{L}}_0$ is a list that is valid as input to $\mathrm{F}_1^{(m)}(\mathrm{G}_0, \vec{\mathbf{L}}_0, \vec{\mathbf{S}})$. This requires that every vertex appears no more than once in $\vec{\mathbf{L}}_0$. $\vec{\mathbf{L}}_1(\vec{\mathbf{L}}_0)$ maps the list $\vec{\mathbf{L}}_0$, which contains vertices from G_0 to a list that contains vertices is $\mathrm{G}_1(\mathrm{G}_0)$ by removing the second subscript in every vertex e.g. if $\vec{\mathbf{L}}_0 = (\{\mathrm{v}_{12}, \mathrm{v}_{24}\}, \{\mathrm{v}_{23}, \mathrm{v}_{33}\}, \ldots)$, $\vec{\mathbf{L}}_1(\vec{\mathbf{L}}_0) = (\{\mathrm{v}_1, \mathrm{v}_2\}, \{\mathrm{v}_2, \mathrm{v}_3\}, \ldots)$.

Lemma 5. *$nLCC(F_n^{(m)}(G_1(G_0), \vec{\mathbf{L}}_1(\vec{\mathbf{L}}_0), \vec{\mathbf{S}})) \geq LCC(F_1^{(m)}(G_0, \vec{\mathbf{L}}_0, \vec{\mathbf{S}}))$ for any valid list $\vec{\mathbf{L}}_0$.*

Proof. For $\vec{\mathbf{L}}_0$ to be a valid input to $\mathrm{F}_1^{(m)}(\mathrm{G}_0, \vec{\mathbf{L}}_0, \vec{\mathbf{S}})$, every vertex appears at most once in it. n vertices from G_0 in $\vec{\mathbf{L}}_0$ map to 1 vertex from G_1 in $\vec{\mathbf{L}}_1(\vec{\mathbf{L}}_0)$. Hence, every vertex appears at most n times in $\vec{\mathbf{L}}_1(\vec{\mathbf{L}}_0)$ and therefore $\vec{\mathbf{L}}_1(\vec{\mathbf{L}}_0)$ is a valid input to $\mathrm{F}_n^{(m)}(\mathrm{G}_1, \vec{\mathbf{L}}_1(\vec{\mathbf{L}}_0), \vec{\mathbf{S}})$.

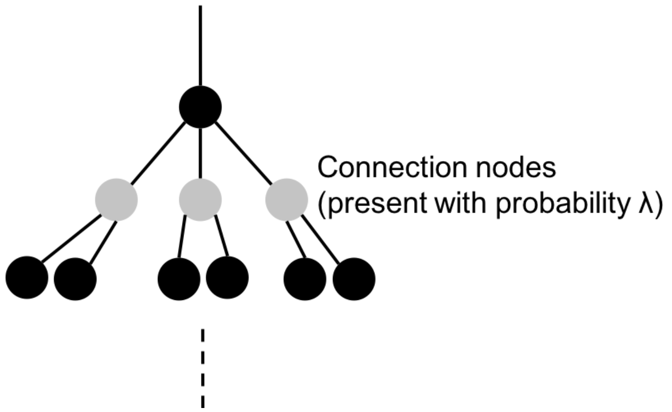

Supplementary Figure 2. Depiction of the expected number of children of a node (black) in Lemma 6 for the case of $n = 4$, $m = 3$. If every connection node (gray) is present with probability λ, the expected number of children after one generation is $\lambda(m-1)(n-1)$.

If two vertices from different clusters $\mathrm{C_a}$ and $\mathrm{C_b}$ in $\mathrm{F}_1^{(m)}(\mathrm{G}_0, \vec{\mathbf{L}}_0, \vec{\mathbf{S}})$ are connected, there must exist at least one element in $\vec{\mathbf{L}}_0$ of the form $\mathrm{L}_{0k} = \{\mathrm{v}_{al}, \mathrm{v}_{br}, \ldots\}$ in $\vec{\mathbf{L}}_0$ with the corresponding entry in $\vec{\mathbf{S}}$, $S_k = 1$. $\mathrm{l}, \mathrm{r}, \ldots \in 1, 2, \ldots, n$. Hence the corresponding entry in $\vec{\mathbf{L}}_1(\vec{\mathbf{L}}_0)$ must have the form $\mathrm{L}_{1k} = \{\mathrm{v}_a, \mathrm{v}_b\}$ with $S_k = 1$. Therefore, if two vertices in two different clusters $\mathrm{C_a}$ and $\mathrm{C_b}$ are connected in $\mathrm{F}_1^{(m)}(\mathrm{G}_0, \vec{\mathbf{L}}_0, \vec{\mathbf{S}})$, the corresponding vertices $\mathrm{v_a}$ and $\mathrm{v_b}$ in $\mathrm{F}_n^{(m)}(\mathrm{G}_1(\mathrm{G}_0), \vec{\mathbf{L}}_1(\vec{\mathbf{L}}_0), \vec{\mathbf{S}})$ are connected. In addition to vertices from G_0, $\mathrm{F}_1^{(m)}(\mathrm{G}_0, \vec{\mathbf{L}}_0, \vec{\mathbf{S}})$ contains vertices created during the operation $\mathrm{F}_1^{(m)}$. If $\mathrm{L}_{0k} = \{\mathrm{v_{al}}, v_{\mathrm{br}}, \ldots\}$ creates a vertex w that is connected to $\mathrm{C_a}$ and $\mathrm{C_b}$ in $\mathrm{F}_1^{(m)}(\mathrm{G}_0, \vec{\mathbf{L}}_0, \vec{\mathbf{S}})$, $\mathrm{L}_{1k} = \{\mathrm{v_a}, \mathrm{v_b}\}$ creates a vertex w that is connected to $\mathrm{v_a}$ and $\mathrm{v_b}$ in $\mathrm{F}_n^{(m)}(\mathrm{G}_1(\mathrm{G}_0), \vec{\mathbf{L}}_1(\vec{\mathbf{L}}_0), \vec{\mathbf{S}})$.

Suppose that the largest connected component of $\mathrm{G_{F1}} = \mathrm{F}_1^{(m)}(\mathrm{G}_0, \vec{\mathbf{L}}_0, \vec{\mathbf{S}})$ contains vertices from k different clusters. Let us call the set of vertices in these clusters $\mathcal{C}$. Since the size of every cluster is n, the number of vertices from G_0 in the largest connected component of $\mathrm{G_{F1}}$ is $\mathrm{LCC}_{\mathrm{G}_0} \leq nk$ with the equality holding if any two vertices within the same cluster are connected. From the previous paragraph, if a vertex of a cluster C_k from G_0 is a part of the largest connected component of $\mathrm{G_{F1}}$, the corresponding vertex v_k from $\mathrm{G}_1(\mathrm{G}_0)$ is part of the largest connected component of $\mathrm{G_{Fn}} = \mathrm{F}_n^{(m)}(\mathrm{G}_1(\mathrm{G}_0), \vec{\mathbf{L}}_1(\vec{\mathbf{L}}_0), \vec{\mathbf{S}})$ and the number of vertices from $\mathrm{G}_1(\mathrm{G}_0)$ in the largest connected component of $\mathrm{G_{Fn}}$ is $\mathrm{LCC}_{\mathrm{G}_1} = k$. In addition to vertices from G_0, the largest connected component of $\mathrm{G_{F1}}$ also contains vertices created during the operation $F_1^{(m)}$. Every vertex not in G_0 that is part of the largest connected component of $\mathrm{G_{F1}}$ comes from an element of $\vec{\mathbf{L}}_0$ that contains a vertex from $\mathcal{C}$ with the corresponding entry of $\vec{\mathbf{S}}$ equal to 1. From the previous paragraph, since every entry of $\vec{\mathbf{L}}_0$ containing a vertex from $\mathcal{C}$ has a corresponding entry in $\vec{\mathbf{L}}_1(\vec{\mathbf{L}}_0)$ that contains a vertex from $\mathrm{G}_1(\mathrm{G}_0)$ corresponding to a cluster in $\mathcal{C}$, the number of vertices in the largest connected component of $\mathrm{G_{F1}}$ not in G_0 is less than or equal to the number of vertices in the largest connected component of $\mathrm{G_{Fn}}$ not in $\mathrm{G}_1(\mathrm{G}_0)$, which we call $\mathrm{LCC_F}$. The equality holds when any two vertices within the same cluster in G_0 are connected. Therefore, $n\mathrm{LCC}(\mathrm{F}_n^{(m)}(\mathrm{G}_1(\mathrm{G}_0), \vec{\mathbf{L}}_1(\vec{\mathbf{L}}_0), \vec{\mathbf{S}})) = n(\mathrm{LCC}_{\mathrm{G}_1} + \mathrm{LCC_F}) = nk + n\mathrm{LCC_F} \geq nk + \mathrm{LCC_F} \geq \mathrm{LCC}_{\mathrm{G}_0} + \mathrm{LCC_F} = \mathrm{LCC}(\mathrm{F}_1^{(m)}(\mathrm{G}_0, \vec{\mathbf{L}}_0, \vec{\mathbf{S}}))$ which proves Lemma 5. □

Lemma 6. *For $\lambda < 1/[(n-1)(m-1)]$, $LCC(F_n^{(m)}(G_1(G_0), \vec{\mathbf{L}}_2, \vec{\mathbf{S}}(\lambda))) = o(N)$. for any valid list $\vec{\mathbf{L}}_2$ where $\vec{\mathbf{S}}(\lambda)$ is a string of bits with the same number of elements as $\vec{\mathbf{L}}_2$ with every bit randomly and independently chosen as 1 with probability λ and 0 with probability $1-\lambda$.*

Proof. Following the same reasoning as refs. [1, 2], starting at a node, we obtain the largest number of expected children of a node when $\vec{\mathbf{L}}_2$ is in the form of a tree as shown in Supplementary Fig. 2. To obtain an infinitely sized connected component (percolate), the expected number of children after a generation is greater than one. The node can have edges to a maximum of n connection nodes. Excluding the the connection node connecting the node to it's parent, $n-1$ of these connection nodes can result in children. Each connection node is present with probability λ. The presence of a connection node adds $m-1$ children to the node. Hence the expected number of children is $\lambda(n-1)(m-1)$ and the condition for percolation is $\lambda(n-1)(m-1) > 1$ or $\lambda > 1/[(n-1)(m-1)]$. This proves Lemma 6. □

Similar to the previous subsection, lemmas 4-6 lead to Theorem 2.

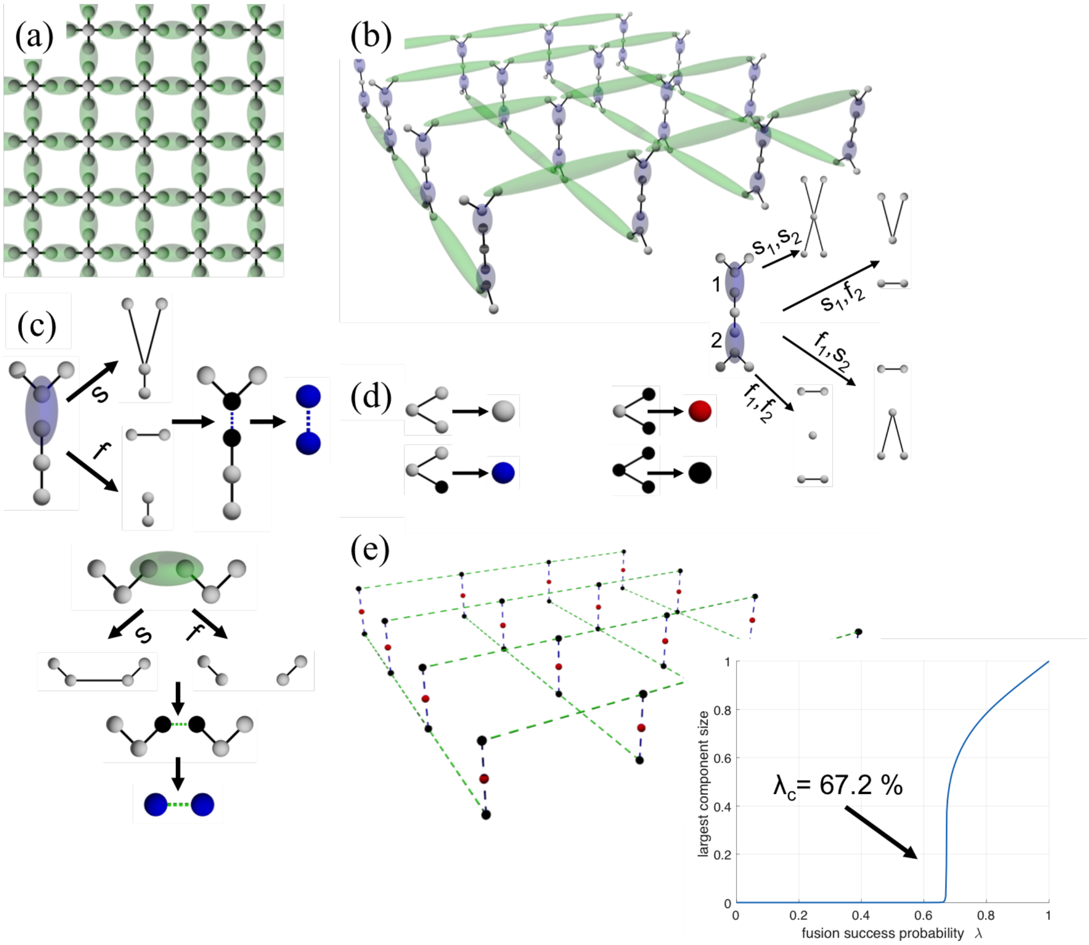

Supplementary Figure 3. Different strategies and logical interpretations of piecing together a 2D square lattice by fusing microclusters: (a) 5-photon microclusters at each lattice node with fusion attempts on each lattice bond; (b) vertical arrangements of three 3-photon microclusters and 2 fusions create a 5-photon cluster if both fusions succeed; (c) interpreting fusion as coloring the measured nodes black and drawing a new bond between them if fusion succeeds, the linear optical circuits corresponding to the blue and green ellipses are shown in Supplementary Fig. 2 and 3 of [3] respectively; (d) mapping microclusters to nodes in a logical graph and coloring them based on how many photons in the microcluster are left unmeasured; (e) pure bond percolation on the logical graph of colored nodes.

SUPPLEMENTARY NOTE 2. FUSING MICROCLUSTERS ON A REGULAR LATTICE

In this section, we describe our mapping from fusion operations to logical lattices with the example of a 2D square lattice. The percolation threshold of the logical lattice gives us the minimum success probability of the fusion operation required to obtain a percolated cluster state.

A. A conservative approach: site-bond percolation

We begin by preparing 5-photon clusters in a star topology and placing them at each node of the lattice, as proposed in Ref. [4], and shown in Supplementary Fig. 3(a). Suppose we succeed in preparing each of those clusters with probability q. We then attempt 2-photon fusions across each edge of the lattice, each of which succeeds with probability λ. The resulting graph state that is generated is a random instance of site-bond (mixed) percolation [5] where each bond is occupied with probability $p = \lambda$ and each site is occupied with probability q. The boundary in the (q, p) space that separates the percolating from the non percolating region is shown by the red solid plot in Supplementary Fig. 4(a). We also show an analytical approximation of this critical boundary (blue dash-dotted plot), developed by Tarasevich and van der Marck [6]. If one had 3-photon clusters (GHZ states) as a starting resource, one can assemble a 5-photon star by attempting two fusions on three 3-photon clusters, as shown in Supplementary

Fig. 3(c). The probability of success in creating the 5-node star is thus $q = \lambda^2$, the probability that both fusions succeed. If either fusion fails, we call it a node failure. Therefore, per Kieling *et al.*'s recipe, the threshold value of λ beyond which one gets percolation is given by the intersection of the site-bond critical boundary and the line $q = p^2$, thereby obtaining $\lambda_c \approx 0.825$ (see Supplementary Fig. 4(a)).

B. Exploiting failure modes: modified site-bond percolation with two stuck-open layers

It is too conservative to ask for both fusions to succeed at every node [3]. In other words, even if one or both fusions in creating the 5-node star were to fail, the leftover cluster fragments can still provide some connectivity. We illustrate this in Supplementary Fig. 3(b), where we lay out the three 3-photon clusters at each node of the square lattice in the vertical arrangement shown, while the square lattice is divided into two crisscrossing layers of parallel 1D lattices. It is as if the lattice is stuck open at each node. If both fusions at a node—shown as light blue ovals—succeed (which happens with probability $q = \lambda^2$), the photon at the center of the vertical arrangement gets attached to the two photons in the top layer as well as the two in the bottom layer, thus forming the 5-photon star. This has the effect of connecting the two layers at that node. If one or both fusions at a node fail (with probability $1 - q$), the node remains stuck open. But, even so, the two nodes in the top layer of the vertical arrangement remain connected to one another, and the same is true for the two nodes in the bottom layer. If one of the two fusions in the vertical arrangement succeeds (and the other fails), the two nodes in the layer closer to the successful fusion are connected via the center node, whereas the two nodes in the other layer (one closer to the failed fusion) are connected to one another directly. In all of these cases (i.e., if one or both fusions fail), the middle node plays no role in terms of providing long-range connectivity. The green ovals show fusion attempts between adjacent nodes in each of the two layers, the bonds of the square lattice.

We begin with an illustrative example of piecing together a large subgraph of the 2D square lattice by probabilistic fusion of microclusters using two-photon destructive fusion operations that succeed with probability λ.

The situation looks identical to (q, p) site-bond percolation with $q = \lambda^2$ and $p = \lambda$, except that even if a site is not active, the four neighboring bonds at that site can be pairwise connected to one another in the two stuck-open layers. We numerically evaluated the percolation region of this modified site-bond problem using the Newman-Ziff algorithm [7] on a square lattice of 1 million nodes. The resulting percolation boundary is shown in the magenta dashed plot in Supplementary Fig. 4(a). This intersects with $q = p^2$ at $p = \lambda \approx 0.672$. This threshold is already below 0.78125, the success probability attainable by linear-optical fusion boosted with ancilla single photons [8].

C. Pure bond percolation on a logical graph

Let us revisit the picture in Supplementary Fig. 3(b), and consider a new interpretation where each 3-photon cluster is thought of as a single (super) node in a logical graph shown in Supplementary Fig. 3(e). We assign a color to the super node based on how many of its photons are intended to be measured (and hence destroyed) in the planned fusion attempts (Supplementary Fig. 3(d)). The central photons in the 3-photon clusters at the centers of the vertical arrangements in Supplementary Fig. 3(b) are not measured as part of a fusion. Hence, those 3-photon clusters map to a red node in the logical graph in Supplementary Fig. 3(e). All other 3-photon clusters in Supplementary Fig. 3(b) will have all their three photons measured in fusion operations and so, all these 3-photon clusters are represented as black nodes in the logical graph. In the logical graph, a node represents an n-photon cluster, and a node's degree equals the number of its photons that will be measured in fusion attempts (and hence destroyed). A bond in the logical graph represents a fusion attempt, which is successful with probability λ. With this new interpretation, the modified site-bond percolation discussed above can now be seen as simple single-parameter bond percolation on the logical graph, where each bond is independently activated with probability λ. It is simple to verify numerically (see the plot in Supplementary Fig. 3(e)) that the bond percolation threshold equals $\lambda_c \approx 0.672$, as expected.

Let us say there are N unmeasured photons in the lattice in Supplementary Fig. 3(b). It is clear from the figure that there are $8N$ photons that get measured, making it a total of $9N$ photons. The logical graph in Supplementary Fig. 3(e) has $3N$ nodes, where each node represents a 3-photon (GHZ) cluster.

Each bond of the logical graph G represents a fusion attempt. All the photons within the black nodes disappear after the fusion attempts have been made but help provide long-range connections, if more than λ_c fraction of the bonds (fusion attempts) were successful. Only the red nodes, which in the example of Supplementary Fig. 3(e) contain a single photon each after the fusion attempts have been made, remain as part of the resulting connected component(s) comprised of the unmeasured photons in the red nodes.

If the individual fusions succeed with probability, $\lambda > \lambda_c$, the bond percolation threshold of F, with high probability, the largest connected component (LCC) will comprise of $O(N)$ unmeasured photons, also known as giant connected

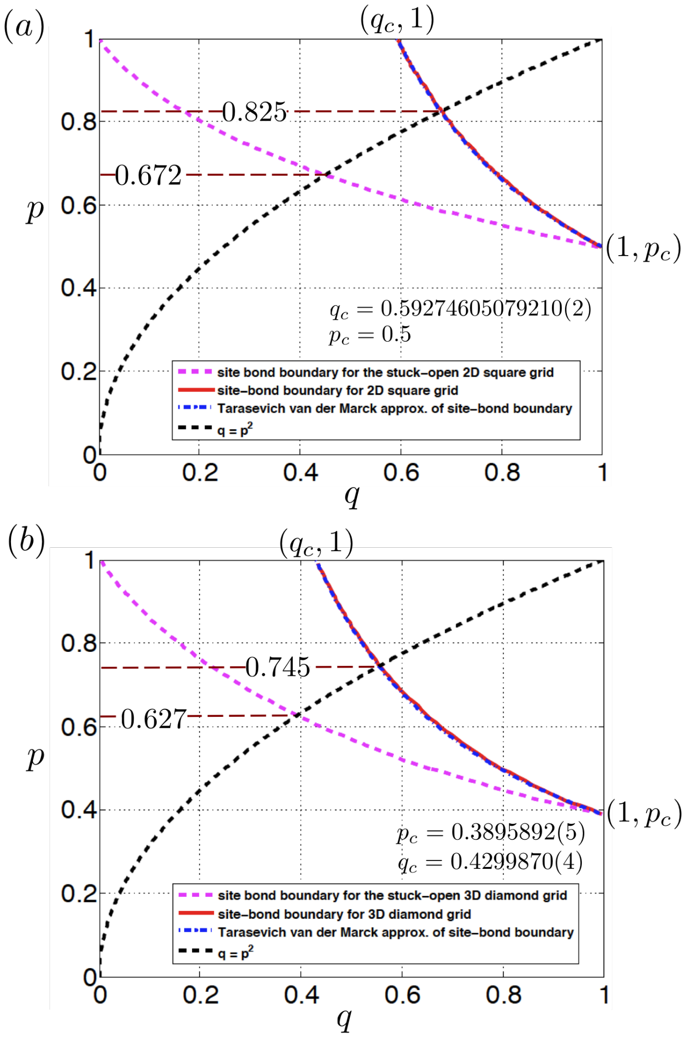

Supplementary Figure 4. Site-bond percolation critical boundaries shown for the (a) 2D square and (b) 3D diamond lattices. The magenta curves correspond to a modified site-bond percolation problem described in the text where even if a site is not occupied, neighboring bonds can still be pairwise connected if occupied.

component (GCC). The constant in this $O(N)$ is given by the y-axis in the plot in Supplementary Fig. 3(e). This GCC is subsequently renormalized into a perfect square grid of $O(N)$ nodes, which is used for cluster-model quantum computing.

These $O(N)$ nodes have both red nodes and black nodes. However, since every black node has a red node at a constant distance (one bond away) from it, the probability of a red node being connected to the nearest black node is a constant. Therefore, the presence of $O(N)$ black nodes in the GCC implies the presence of $O(N)$ red nodes in the GCC, and hence, there must be $O(N)$ red nodes in the GCC whenever there are $O(N)$ nodes in the GCC.

Finally, note that in the example shown in Supplementary Fig. 3(e), even though the logical graph—which describes how to lay out the microclusters prior to fusion attempts—is a non-planar two-layer graph, the physical giant cluster (of the unmeasured photons) obtained from percolation is a subgraph of the planar square lattice.

SUPPLEMENTARY NOTE 3. EVIDENCE IN SUPPORT OF CONJECTURE THAT THE $\lambda_c^{(n)} \geq 1/(n-1)$ BOUND IS TIGHT

We provide two pieces of evidence which support the conjecture that our bound of $\lambda_c^{(n)} \geq 1/(n-1)$ is achievable.

We first present the construction of a graph, starting with n photon clusters, with λ_c approaching $1/(n-1)$. This graph is a tree, and hence not a resource for universal quantum computing, but it may be possible to construct finite dimensional lattices approaching this three in future work with a percolation threshold approaching $1/(n-1)$, similar

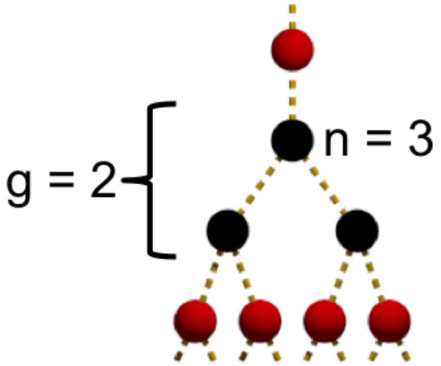

Supplementary Figure 5. Schematic of the lattice construction used to approach the $\lambda_c = 1/(n-1)$ limit for the case of $n = 3$ and $g = 2$.

to the high dimensional extension of the (10,3)-b lattice.

The second piece of evidence is a construction of a random graph that may saturate the $1/(n-1)$ bound with the correct fusion operation.

A. Construction of a tree graph with λ_c approaching $1/(n-1)$

The logical graph that can be used to approach the $1/(n-1)$ limit is shown in Supplementary Fig. 5, for the case of $n = 3$. We start the depiction of our tree at a degree $n-1$ unmeasured node (i.e., a node with an unmeasured qubit), after which there are g generations of degree n black nodes, followed by a generation of unmeasured nodes, followed by g generations of black nodes and so on. In the tree depicted in Supplementary Fig. 5, $g = 2$. Starting from an unmeasured node, given a bond probability of λ, the expected number of unmeasured nodes after $g+1$ generations is $\lambda(n-2)[\lambda(n-1)]^g$. Hence the critical bond percolation probability must satisfy $\lambda_c(n-2)[\lambda_c(n-1)]^g = 1$, which gives us $\lambda_c = (n-2)^{-1/(g+1)}(n-1)^{-g/(g+1)}$. As g increases, we approach the limit of $\lambda_c = 1/(n-1)$. In the argument above, we only count the number of unmeasured nodes and condition (a) of SUPPLEMENTARY NOTE 3 B is satisfied.

In the construction of the lattice above, the input states are n photon cliques, which are equivalent, up to local operations, to n photon GHZ states. The fusion operation used here (dashed lines), acting on two qubits A and B, consists of a Hadamard gate on qubit A followed by Bell measurement of A and B in the $\{1/\sqrt{2}(|00\rangle \pm |11\rangle), 1/\sqrt{2}(|01\rangle \pm |10\rangle)\}$ basis (also described in [9]). Since the order of the Bell measurements is not important, we imagine first applying the fusion operations corresponding to successes. A successful fusion between two cliques removes qubits A and B from the graph and places the rest of the photons in a clique. Hence any two logical nodes that have an edge in Supplementary Fig. 5 are part of the same clique and hence connected. A failed fusion results in an X measurement on A and a Z measurement on B. The Z measurement of a qubit simply removes the photon and all its edges. The X measurement of a qubit in a clique removes the measured qubit and all its edges, and applies a Hadamard gate to one of the qubits that neighbored the measured qubit (before it was removed). This can be seen by looking at the set of stabilizers obtained when an a qubit in a clique is measured in the X basis as detailed in [9]. Since a Hadamard gate followed by a Z (resp., X) measurement has the effect of an X (resp., Z) measurement, the result of the failed fusions is simply the removal of the corresponding nodes from the cliques without disturbing the connectivity between any other nodes. Hence the fusion operation described here can be used to create the logical graph in Supplementary Fig. 5.

B. Random graphs

The discussion in SUPPLEMENTARY NOTE 2 logically leads to a new approach to constructing a large percolated network of photons for ballistic LOQC. We directly pick an N-node logical graph G each node of which represents an n-photon microcluster. We color the nodes based on how many photons we intend to leave unmeasured, or equivalently, the node's intended degree in the logical graph. If the node's degree is d, $1 \leq d \leq n$, we give it color $n-d$, the number of photons in the microcluster at that node that are left unmeasured. See Supplementary Fig. 3(d). The goal is, given n, to pick a logical graph and node coloring such that one gets the lowest possible bond percolation

threshold. In addition, for the percolated cluster to be useful for QC, one must be able to argue that: (a) there are $O(N)$ non-zero-color nodes in the GCC, and (b) every cycle in G has at least one node of non-zero color.

Let us assume G is a regular lattice with uniform node degree d. Let us also assume that we have access to d-node microclusters. One strategy to pick the nodes in G which are to be designated to have non-zero-color is to pick a random α fraction of the N nodes in G as color-1 and populate them with d-photon star clusters. Clearly, these nodes will have one less degree $(d-1)$. We then populate d-photon clique clusters at the remaining $(1-\alpha)N$ degree-d nodes. These nodes have color 0 and hence all the photons in the cliques will get measured up in the fusions. It appears now that if α is small, then the fusion success probability exceeding the bond percolation threshold of G, i.e., $\lambda > p_{\mathrm{c}}(G)$, should be sufficient to guarantee percolation. This would mean that $\lambda_{\mathrm{c}}^{(n)} \leq \min_{G(V,E):deg(V)=n} p_{\mathrm{c}}(G)$. In order to prove this formally, one would need to argue that the conditions (a) and (b) in the previous paragraph are met. We leave this for future work. If this conjecture was proven true, given that the bond percolation threshold of the degree-3 3D (10,3)-b lattice is 0.546694 [10], it would mean that $\lambda_{\mathrm{c}}^{(3)} \leq 0.546694$ for a 3D lattice. Furthermore, it is possible to generalize the (10,3)-b lattice to higher dimensions, following a procedure similar to the generalization of the "modified" (10,3)-b lattice in subsection "Improved thresholds" in the "Results" section of the main paper. Under this construction, $\lambda_{\mathrm{c}} \to 0.5$ as the number of dimensions $\to \infty$. Combined with our converse of $\lambda_{\mathrm{c}}^{(3)} \geq 0.5$, this would imply that $\lambda_{\mathrm{c}}^{(3)} = 0.5$. We conjecture that a higher dimensional construction with size $n \geq 3$ microclusters can saturate the converse bound which would imply that $\lambda_{\mathrm{c}}^{(n)} = 1/(n-1)$, $\forall n \geq 3$. A schematic of the setup described in the discussion above, with G chosen as the 2D square lattice for illustration, is shown in Supplementary Fig. 6.

[1] Broadbent, S. R. & Hammersley, J. M. Percolation processes. *Mathematical Proceedings of the Cambridge Philosophical Society* **53**, 629–641 (1957).

[2] Sahimi, M., Hughes, B. D., Scriven, L. E. & Davis, H. T. On Polya random walks, lattice Green functions, and the bond percolation threshold. *J. Phys. A: Math. Gen.* **16**, 67–72 (1983).

[3] Gimeno-Segovia, M., Shadbolt, P., Browne, D. E. & Rudolph, T. From Three-Photon Greenberger-Horne-Zeilinger States to Ballistic Universal Quantum Computation. *Physical Review Letters* **115**, 020502 (2015).

[4] Kieling, K., Rudolph, T. & Eisert, J. Percolation, Renormalization, and Quantum Computing with Nondeterministic Gates. *Physical Review Letters* **99**, 130501 (2007).

[5] Hammersley, J. M. A generalization of McDiarmid's theorem for mixed Bernoulli percolation. *Mathematical Proceedings of the Cambridge Philosophical Society* **88**, 167 (1980).

[6] Tarasevich, Y. Y. & van der Marck, S. C. An investigation of site-bond percolation on many lattices. *International Journal of Modern Physics C* **10**, 14 (1999).

[7] Newman, M. E. J. & Ziff, R. M. Fast Monte Carlo algorithm for site or bond percolation. *Physical Review E - Statistical, Nonlinear, and Soft Matter Physics* **64**, 1–16 (2001).

[8] Ewert, F. & van Loock, P. 3 / 4 -Efficient Bell Measurement with Passive Linear Optics and Unentangled Ancillae. *Physical Review Letters* **113**, 140403 (2014).

[9] Zaidi, H. A., Dawson, C., Van Loock, P. & Rudolph, T. Near-deterministic creation of universal cluster states with probabilistic Bell measurements and three-qubit resource states. *Physical Review A - Atomic, Molecular, and Optical Physics* **91**, 042301 (2015).

[10] Tran, J., Yoo, T., Stahlheber, S. & Small, A. Percolation thresholds on three-dimensional lattices with three nearest neighbors. *Journal of Statistical Mechanics: Theory and Experiment* **2013**, P05014 (2013).

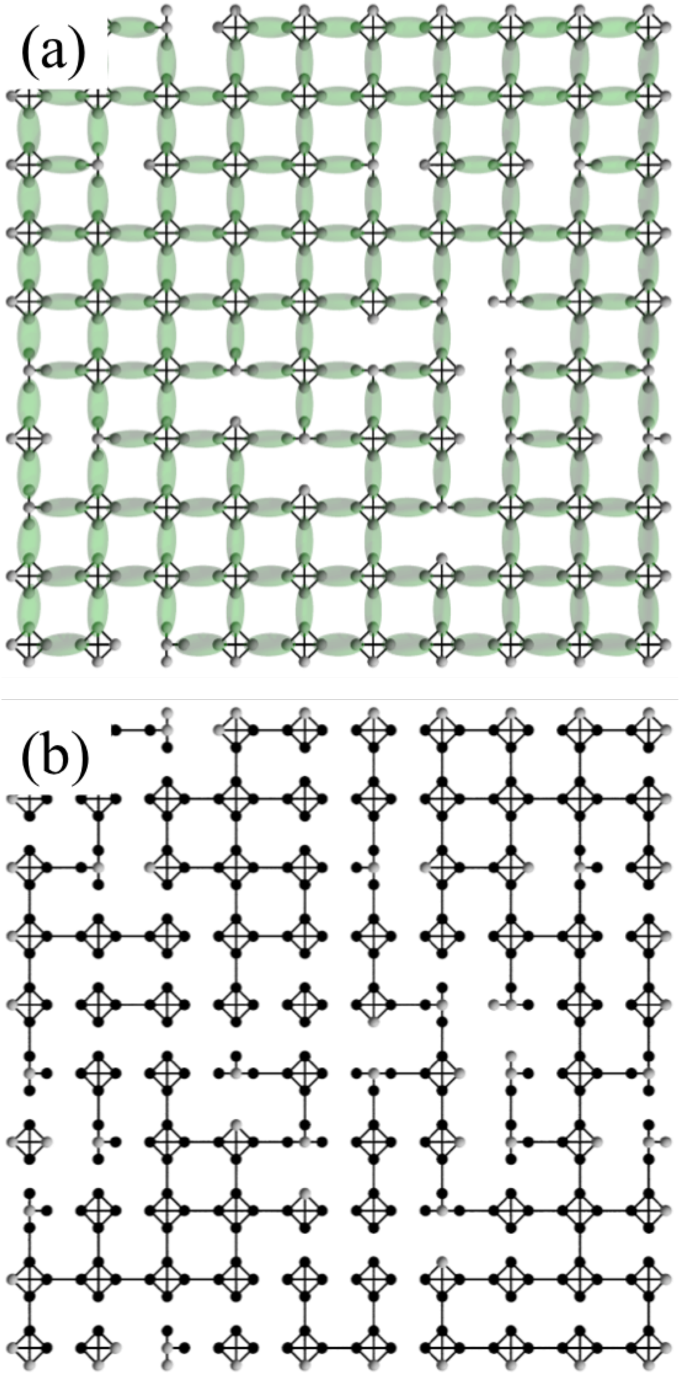

Supplementary Figure 6. (a) 4-node microclusters laid out on nodes of a square lattice. A random $\alpha = 0.3$ fraction of microclusters are put in star configuration the central photon of which will not be measured in any fusion operation. All other photons are measured in fusion attempts. (b) A random instance after the fusion attempts, assuming that each fusion succeeds with probability $\lambda = 0.6$. The measured photons are colored black. The unmeasured photons (colored white) in the giant component of the percolated lattice form the backbone random graph that is renormalized into a fully connected 2D topology for universal cluster-state quantum computing.
